# Three-dimensional *in vitro* models for assessing temozolomide sensitivity in glioblastoma

**DOI:** 10.3389/fnins.2026.1901494

**Published:** 2026-10-21

**Authors:** Andresa Hiromi Sakai, Marna Eliana Sakalem, Luiza Magosso Oliveira Rocha, Isabely Mayara da Silva, Flávia Eliza Staut Silva, Diego Luís Ribeiro, Carlos Frederico Martins Menck, Helber Alves Negreiros, João Marcelo de Castro e Sousa, Juliana Mara Serpeloni

**Affiliations:** 1 Department of General Biology, Center of Biological Sciences, State University of Londrina (UEL), Londrina, Brazil; 2 Department of Anatomy, Center of Biological Sciences, State University of Londrina (UEL), Londrina, Brazil; 3 Department of Microbiology, Institute of Biomedical Sciences, University of São Paulo (ICB/USP), São Paulo, Brazil; 4 Postgraduate Program in Pharmaceutical Sciences, Toxicological Genetics Research Laboratory, Federal University of Piauí, Teresina, Brazil; 5 Toxicological Genetics Research Laboratory (Lapgenic), Center for Heath of Sciences, Federal University of Piaui, Teresina, Brazil

**Keywords:** chemoresistance, glioblastoma multiforme, organoids, spheroids, temozolomide

## Abstract

IDH-wildtype glioblastoma, or glioblastoma multiforme (GBM), is the most aggressive primary malignancy of the central nervous system. It is characterized by marked invasiveness, intratumoral heterogeneity, and high recurrence rates, largely driven by intrinsic and acquired resistance to standard temozolomide (TMZ) therapy. TMZ is an oral alkylating agent that methylates nitrogenous bases, mainly forming O^6^-methylguanine (O^6^-meG) lesions, the main cytotoxic and mutagenic events responsible for its antitumor effects. This review first characterizes gliomas and the mechanism of action of the main GBM treatment drug (TMZ), then critically examines *in vitro* cell culture models investigating these mechanisms. This review emphasizes the development and application of three-dimensional (3D) culture systems, including tumor spheroids and organoids, which more effectively recapitulate tumor architecture, cell–cell interactions, and drug penetration gradients than conventional two-dimensional models. This review discusses the strengths and limitations of these models and their translational relevance to modeling TMZ sensitivity and resistance in GBM.

## 1 Introduction

Brain and other central nervous system (CNS) cancers cause approximately 248,000 deaths annually worldwide, and constitute a globally significant cause of cancer mortality (Kim et al., 2025). Although they represent between 1 and 3% of all human neoplasms, they account for approximately 3 to 5% of cancer-related deaths (Louis et al., 2021; Ostrom et al., 2023). In the United States of America, an estimated 24,800 new cases and 18,350 deaths are projected for 2026 (Siegel et al., 2026).

Gliomas are the most common primary CNS tumors, accounting for about 80% of malignant brain tumors and 26.3% of all primary CNS tumors (Ostrom et al., 2023). The World Health Organization (WHO) classification (Louis et al., 2021) groups gliomas into grades I to IV based on histopathological features and molecular characteristics; grade I gliomas present low proliferative potential, whereas grades II to IV are considered diffuse, malignant, and highly invasive. Notably, the current WHO classification integrates molecular parameters as essential diagnostic criteria, rather than adjunct features.

Glioblastoma multiforme (GBM) is the most aggressive and invasive primary malignant brain tumor in adults and is classified as a grade IV diffuse astrocytic tumor according to the WHO CNS5 classification. It represents approximately 45–50% of malignant primary brain tumors, with a higher incidence observed between 60 and 80 years of age (Ostrom et al., 2023). In the current WHO classification, glioblastoma is defined by the absence of isocitrate dehydrogenase (IDH) mutations (IDH-wildtype), whereas IDH-mutant tumors are classified as astrocytomas, grades II–IV. Therefore, in this review, GBM refers specifically to IDH-wildtype glioblastoma, in accordance with contemporary diagnostic criteria (Stoyanov et al., 2022).

The current standard of care for patients with newly diagnosed GBM consists of maximal safe surgical resection followed by radiotherapy with concomitant and adjuvant temozolomide (TMZ), as established by the Stupp protocol (Stupp et al., 2005; Baik et al., 2023; Nabian et al., 2024), which will mark its 20th anniversary in 2026 (Khasraw et al., 2026). According to the SNO/EANO consensus, the standard regimen for patients with adequate performance status includes daily TMZ concurrent with radiotherapy, followed by adjuvant TMZ, typically for six or more cycles. However, treatment may be individualized according to patient age, performance status, *MGMT* promoter methylation status, and other clinical and molecular characteristics (Wen et al., 2020). As an oral alkylating agent, TMZ provides the greatest clinical benefit among systemic therapies for newly diagnosed GBM, partly because of its favorable oral bioavailability, hydrophilic properties, and ability to cross the blood–brain barrier (Wick et al., 2018). Nevertheless, despite advances in multimodal treatment, GBM remains a highly lethal disease, with less than 5–7% of patients surviving beyond 5 years (Ostrom et al., 2023). Moreover, a recent regional study in Spain reported a median overall survival (OS) of 12.3 months for patients with GBM, compared with 38.4 months for those with IDH-mutant astrocytoma (Encarnación et al., 2025). These poor outcomes are largely attributable to the aggressive and infiltrative nature of GBM, as well as the frequent development of therapeutic resistance and tumor recurrence.

The clinical benefit of TMZ varies among patients with GBM due to differences in drug sensitivity. Multiple tumor-intrinsic mechanisms influence TMZ response, including MGMT-mediated repair of O^6^-methylguanine lesions, mismatch repair proficiency, DNA damage response pathways, cell-cycle regulation, and stem-like cellular states (Miramova et al., 2024; Karve et al., 2024; Pu et al., 2025). Microenvironmental factors, such as hypoxia, extracellular matrix composition, cell–cell interactions, and limited drug penetration, may further modulate TMZ sensitivity (Pu et al., 2025). Understanding these interacting factors is essential to elucidate TMZ resistance and develop experimental models that better reflect clinically relevant responses.

Given these limitations, we must further explore the molecular pathophysiology of GBM and develop experimental models that more accurately recapitulate tumor biology. Animal models only partially recapitulate the complexity of human disease, and traditional two-dimensional (2D) cell cultures fail to capture tissue architecture, cellular heterogeneity, and microenvironmental interactions. In this context, three-dimensional (3D) culture systems have emerged as a powerful platform to bridge this gap. Multicellular tumor spheroids and organoids represent the main 3D *in vitro* models used to study brain tumors. Organoids can be generated from patient-derived tumor samples (patient-derived organoids, PDOs) or from induced pluripotent stem cells (iPSCs), enabling the modeling of both tumor-intrinsic and developmental features (Antonica et al., 2022).

In this context, the choice of experimental model critically determines how researchers measure and interpret TMZ sensitivity (Riess et al., 2021; Fabro et al., 2022). Conventional 2D cultures remain valuable for mechanistic studies and large-scale screening but do not reproduce several spatial and microenvironmental features that influence drug response in tumors (Jensen and Teng, 2020; Diao et al., 2019; Hill et al., 2021). Conversely, *in vivo* models provide greater physiological complexity but are limited by cost, throughput, species-specific differences, and reduced experimental accessibility (da Hora et al., 2019; Robertson et al., 2019). 3D systems occupy an important intermediate position, allowing tumor architecture, cell–cell and cell–matrix interactions, oxygen and nutrient gradients, cellular heterogeneity, and drug penetration to be modeled under experimentally controlled conditions. However, considerable heterogeneity exists among 3D GBM platforms in cellular source, culture methodology, TMZ concentration and exposure schedule, and criteria used to define therapeutic response, complicating cross-study comparisons.

Therefore, this review critically examines how 3D *in vitro* models have been used to investigate TMZ sensitivity and chemoresistance in GBM, with particular emphasis on the molecular and microenvironmental determinants of response, differences among experimental platforms, methodological limitations, and opportunities for standardization and improved translational relevance. We conducted a PubMed literature search for pertinent studies published between January 2020 and February 2026. Eligible studies met the following criteria: (i) original research articles, (ii) published in English, (iii) description of the 3D model, (iv) TMZ concentrations reported for the 3D model, (v) treatment duration reported, (vi) use of human cells, and (vii) publication after 2020. We selected 96 studies, and Supplementary Table 1 summarizes their main experimental characteristics and findings.

## 2 The pathophysiology of adult-type diffuse gliomas and glioblastomas

The 2021 5th edition of the WHO Classification of Central Nervous System Tumors (WHO CNS5) established an integrated diagnostic framework that combines histological features, immunohistochemistry, and molecular genetics rather than relying solely on morphology. According to this classification, adult-type diffuse gliomas are categorized into three main classes: (i) astrocytoma, IDH-mutant; (ii) oligodendroglioma, IDH-mutant and 1p/19q-codeleted; and (iii) GBM, IDH-wildtype (Louis et al., 2021) (Figure 1).

**Figure 1:**
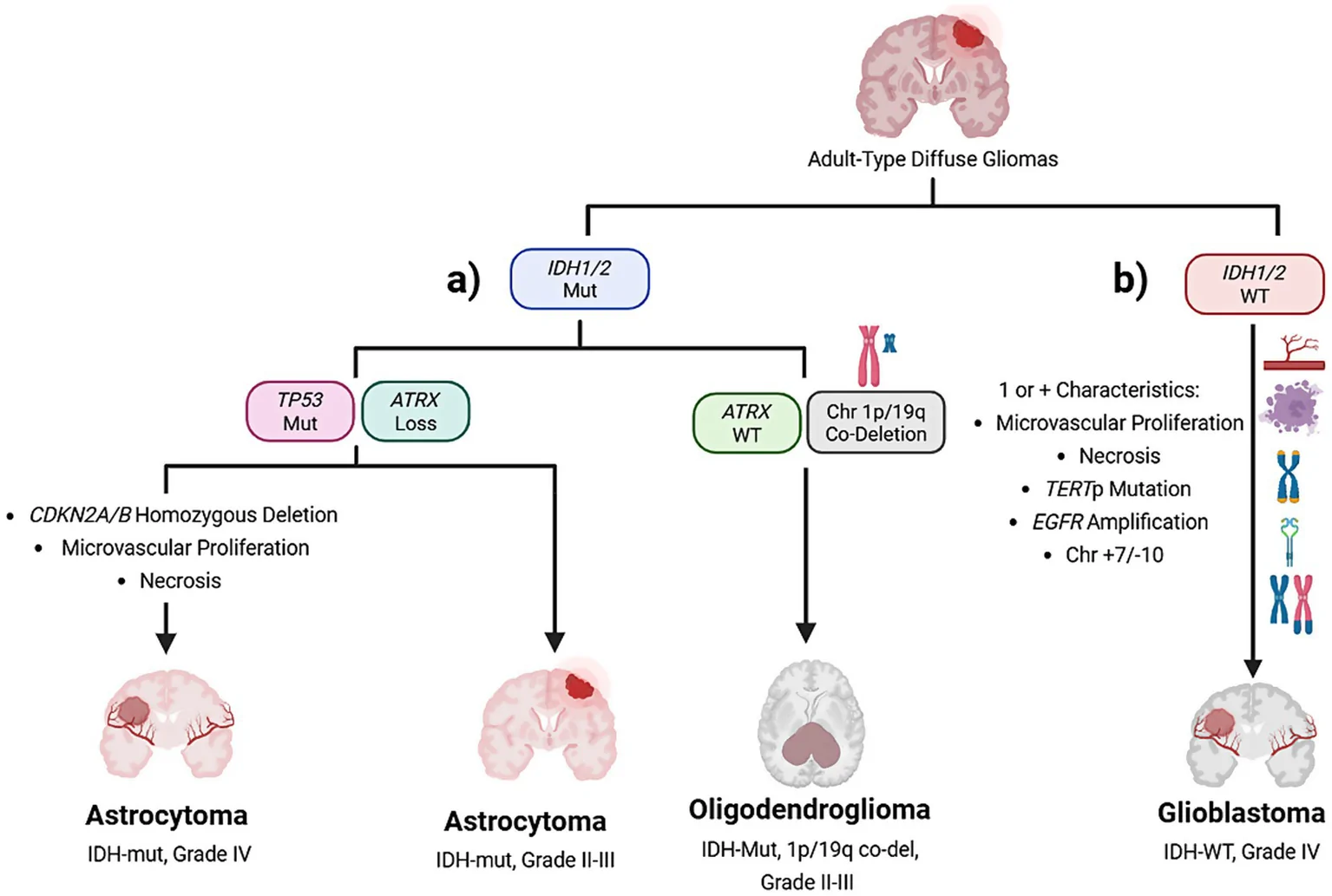
Adult-type diffuse glioma classification integrates histological tumor typing and grading with molecular marker analyses. **(a)** *IDH1/2*-mutant tumors; **(b)** *IDH1/2* wild-type tumors.

Tumors harboring IDH1 or IDH2 mutations are classified as astrocytomas or oligodendrogliomas (Figure 1a). IDH-mutant astrocytoma represents a single entity encompassing CNS WHO grades II–IV, characterized by diffusely infiltrative astrocytic tumors. These tumors typically exhibit Tumor Protein 53 *(TP53)* mutations and loss of Alpha Thalassemia/Mental Retardation Syndrome X-linked *(ATRX)* expression, frequently accompanied by p53 overexpression. Importantly, homozygous deletion of Cyclin-Dependent Kinase Inhibitor 2A/Cyclin-Dependent Kinase Inhibitor 2B *(CDKN2A/CDKN2B)* is sufficient to define WHO grade IV in these tumors, even without classical histological features such as necrosis or microvascular proliferation, highlighting the importance of molecular criteria in current classification (Louis et al., 2021).

Oligodendroglioma, *IDH*-mutant and 1p/19q-codeleted, requires combined whole-arm deletions of chromosomes 1p and 19q for diagnosis. These tumors typically retain *ATRX* expression and frequently harbor Telomerase Reverse Transcriptase *(TERT)* promoter mutations, distinguishing them from astrocytic tumors. Overall, tumors with *IDH* mutations combined with *TP53* and *ATRX* alterations exhibit astrocytic differentiation, whereas those with *IDH* mutation and 1p/19q codeletion display oligodendroglial features (Reuss, 2023).

GBM, defined as an IDH-wildtype diffuse astrocytic tumor, WHO grade IV, represents the most aggressive and invasive primary malignant brain tumor in adults. The diagnosis is established by the presence of histological key features, including necrosis, microvascular proliferation, and specific molecular alterations, such as epidermal growth factor receptor (*EGFR*) amplification, combined with whole chromosome 7 gain and chromosome 10 loss (+7/−10), or *TERT* promoter mutation, even in the absence of classical histological features (Figure 1b) (Al Ghafari et al., 2025). In the WHO CNS5, the term “glioblastoma” is used only for IDH-wildtype tumors (Reuss, 2023).

GBM accounts for approximately 15% of all primary brain tumors and nearly 45–50% of malignant brain tumors (Alorfi et al., 2024). It predominantly affects older adults, with peak incidence between 65 and 80 years of age, and is more prevalent in males and individuals of Caucasian ancestry. Despite advances in diagnosis and treatment, GBM remains associated with a dismal prognosis, with 5-year survival rates below 5–7% (Esparza-Salazar et al., 2025). Its highly diffuse and infiltrative growth pattern significantly limits the feasibility of complete surgical resection (Alorfi et al., 2024).

GBM commonly arises in supratentorial regions, particularly the frontal lobe, and often invades multiple lobes; extracranial metastases are rare (Esparza-Salazar et al., 2025). A substantial body of evidence supports the concept that glioma stem-like cells (GSCs) act as tumor-initiating cells, contributing to tumor propagation, plasticity, and therapeutic resistance (Puletti et al., 2026; Lauko et al., 2022; Neftel et al., 2019). These cells can differentiate and dedifferentiate in response to microenvironmental cues, thereby driving intratumoral heterogeneity (Ribeiro et al., 2025). Historically, GBM was subdivided into primary and secondary forms; however, molecular classification has largely superseded this distinction. Tumors previously classified as secondary GBM are now recognized as IDH-mutant astrocytomas, WHO grade IV (Alorfi et al., 2024).

At the transcriptional level, GBM can also be classified into classical, mesenchymal, and proneural subtypes. The classical subtype is typically associated with *EGFR* amplification. The mesenchymal subtype is associated with loss of neurofibromin 1 *(NF1)* and activation of inflammatory pathways. The proneural subtype presents platelet-derived growth factor receptor alpha *(PDGFRA)* alterations and *TP53* mutations, may benefit from Phosphoinositide 3-kinase/Akt protein/Mammalian Target of Rapamycin (PI3K/AKT/mTOR) or DNA damage response-targeted therapies, but responds poorly to conventional treatment (Ribeiro et al., 2025; Verhaak et al., 2010). Other authors also include a neural category, which accounts for 16% of GBM cases and is characterized by neural marker expression (Al Ghafari et al., 2025). This heterogeneity significantly challenges GBM treatment.

Complex interactions between genetic alterations and the tumor microenvironment drive GBM aggressiveness and progression. Tumor-associated inflammation is mediated by cytokines such as interleukin-6 (IL-6), interleukin-1 beta (IL-1β), and tumor necrosis factor alpha (TNF-α), which activate resident microglia and recruit peripheral macrophages (Esparza-Salazar et al., 2025). These tumor-associated macrophages and microglia (TAMs) can adopt either a pro-inflammatory (M1-like) or anti-inflammatory (M2-like) phenotype (Chen et al., 2025), with GBM typically promoting an immunosuppressive M2-like state that supports tumor growth and immune evasion (Alorfi et al., 2024).

Inflammatory signaling also increases blood–brain barrier permeability, facilitating immune cell infiltration and sustaining chronic neuroinflammation (Li et al., 2024). This environment promotes tumor progression, angiogenesis, and resistance to therapy. In parallel, GBM cells undergo extensive metabolic reprogramming characterized by the Warburg effect, favoring glycolysis for Adenosine Triphosphate (ATP) production even in the presence of oxygen, thereby supporting rapid proliferation and survival under hypoxic conditions (Al Ghafari et al., 2025).

The genetic etiology of GBM remains complex and involves alterations in key oncogenic pathways. Frequently altered genes include *EGFR,* where its amplification activates PI3K/AKT/mTOR and Ras protein/Raf protein/Mitogen-activated protein kinase/Extracellular signal-regulated kinase (Ras/Raf/MEK/EKR) pathways to drive proliferation and invasion; phosphatase and tensin homolog *(PTEN),* deleted in approximately 30%; cyclin-dependent kinase 4 (*CDK4*); mouse double minute 2 and 4 (*MDM2/MDM4*), regulators of p53; and *PDGFRA* (Wen et al., 2025). These alterations collectively drive dysregulated cell-cycle progression, apoptosis resistance, and enhanced invasive potential. In addition, MDM2/MDM4 amplification contributes to functional inactivation of *TP53* signaling, further promoting tumor survival (Al Ghafari et al., 2025; Esparza-Salazar et al., 2025).

Epigenetic alterations are also critical in GBM biology. DNA methylation, particularly O6-methylguanine-DNA methyltransferase (*MGMT*) promoter methylation, is a key predictive biomarker of response to temozolomide therapy (Anan et al., 2024; Briceno et al., 2025; Wu et al., 2021). In addition, histone modifications can activate oncogenes and deregulate other key genes, and chromatin remodeling contributes to gene deregulation (Li and Wu, 2017). The epigenetic alterations lead to aberrant DNA repair, thereby promoting tumor survival by activating pro-survival pathways and enhancing drug efflux transporters (Ribeiro et al., 2025).

The etiology of GBM remains incompletely understood, although several risk factors have been proposed. Ionizing radiation is the best-established environmental risk factor (Li et al., 2020; Smith et al., 2025), whereas evidence for smoking, diet, electromagnetic fields, head trauma, heavy metals, and agrochemicals remains limited (Hanif et al., 2017). More recently, higher body mass index, alcohol consumption, and non-steroidal anti-inflammatory drug use have also been investigated as potentially modifiable risk factors (Yoshikawa et al., 2023). Using paired GBM cases and matched non-tumor controls, Wu et al. (2025) developed a blood-based nomogram that identified six independent factors associated with GBM risk prediction: serum chloride, magnesium, high-density lipoprotein cholesterol, uric acid, eosinophils, and basophils. Although these findings suggest the potential value of readily accessible blood-based parameters for GBM risk stratification, their clinical applicability and predictive validity require further evaluation and external validation. Advances in genomic and epigenetic characterization have also identified important prognostic and predictive markers, particularly IDH mutations and MGMT promoter hypermethylation (Gutiérrez-Arroyo et al., 2026; Ribeiro et al., 2025).

Despite increased understanding of the molecular evolution of GBM in recent years, it remains an incurable disease associated with poor survival outcomes (Melhem et al., 2022). Common manifestations include headache, epileptic seizures, focal neurological deficits, cognitive or behavioral changes, and symptoms related to increased intracranial pressure, such as nausea and vomiting (Hansen et al., 2023; Mrowczynski et al., 2021). Headache may be particularly relevant in older patients when it presents with new-onset or atypical features and should be interpreted alongside other neurological findings (Sipos et al., 2025). Because these symptoms are often nonspecific, clinical suspicion of GBM generally requires integration of the patient’s neurological presentation with neuroimaging findings.

Most GBM treatments focus on offering survival mechanisms, but the prognosis is poor due to the common tumor recurrence and resistance to therapy (Singh et al., 2025). Fit patients undergo maximal safe tumor resection, as the extent of resection is critical for prognosis. As mentioned, the post-surgical procedure involves chemoradiotherapy, usually with TMZ, but recurrence is almost inevitable. Stereotactic radiosurgery and whole-brain radiation therapy, for ineligible patients, can also be considered (Singh et al., 2025). However, tumor recurrence is nearly universal, largely due to intrinsic and acquired resistance mechanisms.

Novel approaches, such as targeted therapies (e.g., EGFR inhibitors), immunotherapies, and tumor-treating fields (TTF), have recently been introduced (Singh et al., 2025). Nevertheless, their clinical efficacy remains limited, mainly because tumor heterogeneity and the blood–brain barrier (BBB) restrict drug delivery to the tumor site (Esparza-Salazar et al., 2025).

## 3 Temozolomide: mechanisms of action and chemoresistance

The U.S. Food and Drug Administration (FDA) initially approved TMZ in 1999 for refractory anaplastic astrocytoma in adults and later approved it in 2005 for newly diagnosed adult GBM (Lee, 2016). Since then, the combination of radiotherapy with concurrent TMZ followed by adjuvant TMZ has become the standard of care for newly diagnosed GBM (Stupp et al., 2005). This regimen, known as the Stupp protocol, consists of maximal safe surgical resection followed by radiotherapy with concurrent TMZ and subsequent adjuvant TMZ, typically for six cycles. It is an imidazotetrazine-class prodrug derived from dacarbazine, an alkylating agent generating cytotoxic DNA lesions (Singh et al., 2021) (Figure 2a).

**Figure 2:**
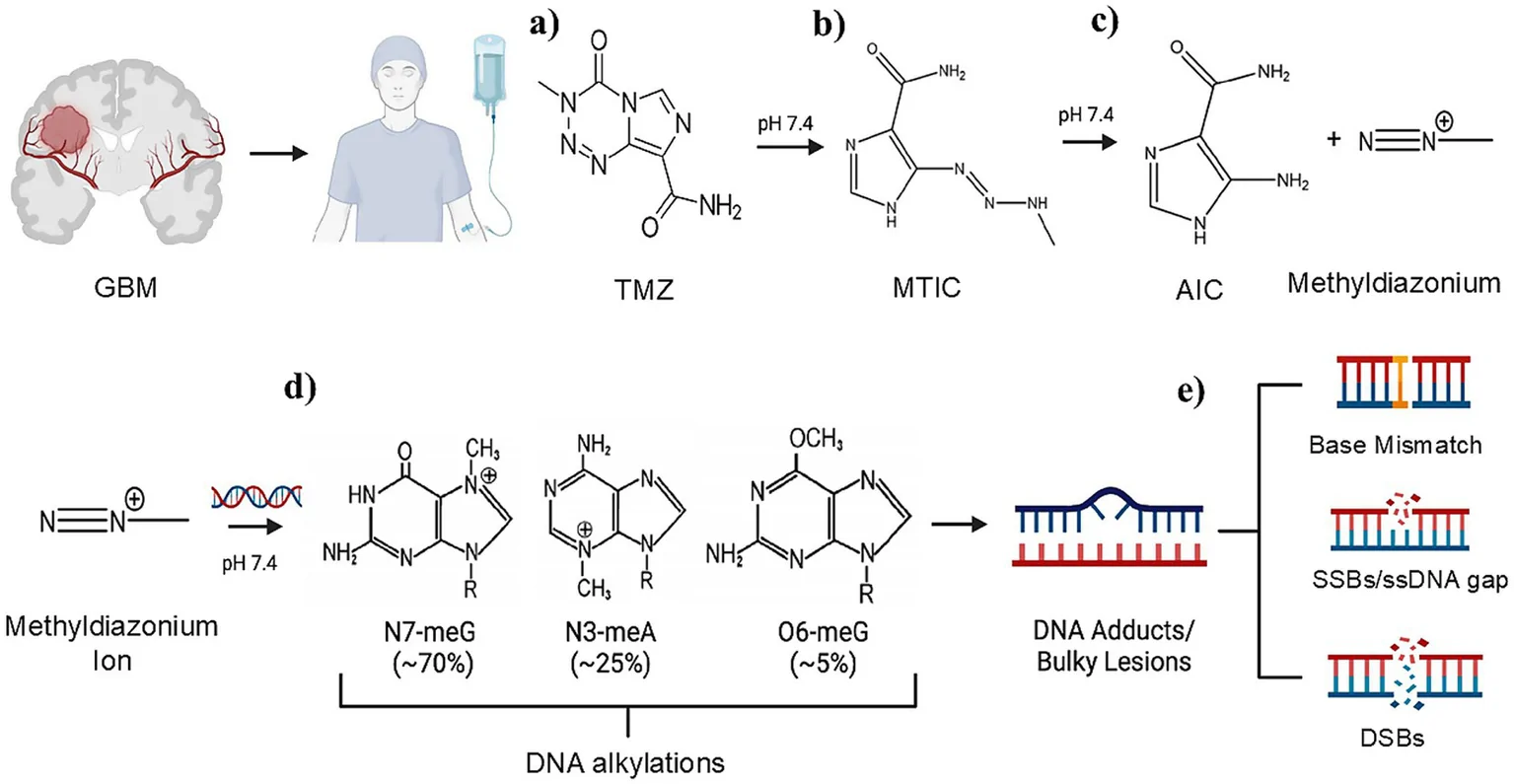
Molecular mechanisms underlying TMZ activity. **(a)** chemical structure of temozolomide; **(b)** hydrolytic conversion to its active metabolite, MTIC; **(c)** decomposition of MTIC to form AIC and the highly reactive methyldiazonium cation; **(d)** methylation of DNA nitrogenous bases, generating genotoxic alkylation lesions; **(e)** accumulation of ssDNA gaps, replication fork stalling and collapse, and the formation of DSBs. GMB, Glioblastoma; TMZ, Temozolomide; MTIC, 5-(3-methyltriazen-1-yl)imidazole-4-carboxamide; AIC, 4-amino-5-imidazole-carboxamide; SSBs, Single strand breaks; ssDNA, single strand DNA; DSBs, double strand breaks.

Following oral administration, TMZ exhibits high bioavailability (>90%) (Gupta et al., 2014). However, although TMZ achieves measurable plasma concentrations after administration, its effective concentrations within the brain tumor microenvironment are substantially lower. Stratenwerth et al. (2021) showed that, despite plasma concentrations ranging from approximately 28 to 72 μmol/L after oral TMZ administration, concentrations in the brain interstitium and cerebrospinal fluid were considerably lower (~1–10 μmol/L), indicating limited drug availability at the target site.

Although TMZ is relatively hydrophilic (LogP ≈ − 1.1), its low molecular weight and neutral chemical structure at physiological pH allow it to penetrate the BBB (Guo et al., 2023). However, TMZ undergoes rapid spontaneous hydrolysis under physiological conditions, leading to a sharp decline in systemic drug levels and resulting in limited concentrations in the brain interstitium and cerebral fluid (Lopes et al., 2013). Clinically relevant intratumoral TMZ concentrations are substantially lower than those commonly used in conventional *in vitro* studies, which often employ supraphysiological TMZ doses (Stepanenko and Chekhonin, 2019).

At physiological pH, TMZ undergoes spontaneous hydrolytic conversion to its active metabolite, 5-(3-methyltriazen-1-yl)imidazole-4-carboxamide (MTIC) (Figure 2b). MTIC subsequently decomposes to form 4-amino-5-imidazole-carboxamide (AIC) and the highly reactive methyldiazonium cation (Lopes et al., 2013) (Figure 2c). The methyldiazonium ion mediates methylation (-CH3) of DNA nitrogenous bases, generating genotoxic alkylation lesions that underlie TMZ’s cytotoxic activity (Figure 2d) (Woo et al., 2019).

TMZ-induced genotoxic lesions primarily include: (i) N7-methylguanine (N7-meG) (~70%), which may undergo depurination, generating abasic sites that impair DNA replication (Boysen et al., 2009); (ii) N3-methyladenine (N3-meA) (~25%), which blocks DNA polymerase progression and can induce mutagenic events, particularly A: T → T: A transversions (Fu et al., 2012); and (iii) O^6^-methylguanine (O^6^-meG) (~5%), the most cytotoxic and clinically relevant lesion, as it mispairs with thymine during replication, initially resulting in G: C → A: T transition mutations (Tentori and Graziani, 2009).

If not removed, O^6^-meG lesions trigger iterative attempts at correction by the mismatch repair (MMR) system, leading to so-called “futile repair cycles.” During DNA replication, O^6^-meG preferentially mispairs with thymine, which is recognized by MutSα MutS Homolog 2/MutS Homolog 6 (MSH2/MSH6) and subsequently processed by downstream MMR components. However, because the methylated guanine remains in the template strand, repeated rounds of excision and resynthesis occur without removal of the lesion (Quiros et al., 2010). These cycles result in the accumulation of single-strand DNA gaps (ssDNA gaps) (Stojic et al., 2004), replication fork stalling and collapse, and ultimately DNA DSB formation (Fuchs et al., 2021) (Figure 2e). Importantly, emerging evidence suggests that post-replicative ssDNA gaps represent critical intermediates underlying TMZ-induced cytotoxicity and therapeutic response (Miramova et al., 2024). The ensuing DNA damage response culminates in cell cycle arrest and cell death (Woo et al., 2019).

However, the consequences of TMZ-induced DNA damage extend beyond acute cytotoxicity. In a subset of tumors, prolonged TMZ exposure can select for tumor cell populations with defects in the MMR pathway, particularly involving MSH6 and other MMR components. When a functional MMR system no longer efficiently recognizes and processes O^6^-meG lesions, cells may escape the lethal consequences of futile repair cycles while continuing to accumulate replication-associated mutations. This can result in a TMZ-associated hypermutator phenotype characterized by a marked increase in somatic mutations, predominantly G > A transitions (Choi et al., 2018). Researchers have observed this hypermutation in recurrent gliomas following TMZ treatment, and it is associated with acquired resistance to further alkylating chemotherapy (Daniel et al., 2019; Touat et al., 2020). This phenomenon has also been described in lower-grade gliomas, where TMZ exposure has been associated with hypermutation during progression to higher-grade disease (Johnson et al., 2026). In a cohort of recurrent IDH-mutant lower-grade gliomas, TMZ-induced hypermutation was identified in 57% of tumors previously exposed to TMZ and was associated with high-grade transformation and reduced survival after transformation (Yu et al., 2021). These findings underscore the complex relationship between TMZ-induced DNA damage, DNA repair capacity, tumor evolution, and therapeutic resistance.

Beyond MMR-mediated processing of O^6^-meG lesions, other DNA repair and damage-tolerance pathways can substantially influence TMZ response. The more abundant N7-meG and N3-meA lesions are primarily repaired through base excision repair (BER), in which poly(ADP-ribose) polymerase 1 (PARP1) facilitates repair of DNA intermediates generated during lesion processing (Miramova et al., 2024; Karve et al., 2024). Homologous recombination (HR) also helps resolve replication-associated DNA damage generated after TMZ exposure, whereas translesion synthesis (TLS) allows DNA replication to proceed across or beyond lesions that would otherwise impair replication (Cheng et al., 2024; Zheng et al., 2025). Increased activity of these repair and tolerance mechanisms can promote cell survival, while their impairment may enhance TMZ sensitivity (Miramova et al., 2024; Latancia et al., 2024). Thus, together with *MGMT* expression and MMR proficiency, the coordinated activity of BER, HR, and DNA damage tolerance pathways contributes to the heterogeneous response of GBM cells to TMZ.

## 4 Common glioblastoma cell lines used in experimental studies

Developing more effective therapeutic strategies for GBM remains a major challenge. In this context, *in vitro* models represent an essential initial step in basic research. Researchers widely use them to improve understanding of the tumor microenvironment and to screen potential therapeutic compounds (Ledur et al., 2017). In particular, GBM cell lines allow the investigation of several mechanisms involved in tumorigenesis, chemoresistance, and DNA damage response pathways. Consider the genotypic, phenotypic, and metabolic differences among cell lines, as these characteristics may significantly influence experimental outcomes. Cell suspensions from different cell lines, seeded at equal numbers to form 3D cultures, may generate spheroids that differ in size, circularity, compactness, and other morphometric parameters (Blondeel et al., 2025), which can directly influence the results, especially in the context of chemoresistance. The types of 3D models usually employed in GBM investigations, along with the strengths and limitations of each, are displayed in Table 1.

**Table 1:** Most commonly used 3D GBM models.

Model	Purpose	Pros	Cons
Scaffold-free			
3D cellular aggregates provide valuable insights into cell–cell interactions, hypoxia core formation, tumor growth, drug responses, and therapeutic resistance challenges			
Spheroids - Ultra-low attachment (ULA)	Spontaneous cell aggregation in suspension via reduced cell-to-plastic adhesion; ideal for high-throughput cytotoxicity screening and investigating core necrosis and hypoxia in GBM cell lines	High reproducibility; low cost; compatible with multiwell plates; suitable for high-throughput screening (HTS); straightforward integration with viability assays	Lacks native brain extracellular matrix (ECM) components; poor spheroid formation and compaction in certain cell lines; variability in size and morphology; limitations in extracellular matrix (ECM) architecture and composition; risk of spheroid loss during fluid handling
Tumorsphere / Glioma Stem Cell (GSC) models	Assessment of cancer stem cell (CSC)-associated features; investigation of stemness traits, including self-renewal, differentiation, anchorage-independent growth, and therapeutic resistance	Enriches for GSC subpopulations reflecting patient intratumoral heterogeneity and driver mutations; evaluation of self-renewal capacity and treatment resistance; reflection of CSC-associated phenotypes; screening of anti-CSC therapeutics; reproduces therapy resistance mechanisms observed in recurrent GBM; ideal for anti-GSC drug investigations	Results are highly sensitive to growth factor depletion; limited spheroid formation in specific cell lines; difficulty evaluating sphere-forming efficiency due to mixed cell populations and aggregation artifacts; central necrosis in larger spheres interferes with cytotoxicity assays
Hanging-drop - Spheroids	Spheroid assembly mediated by gravity and surface tension; cell concentration within small droplet volumes; utilized for generating uniform GBM spheroids or co-culture aggregates (e.g., GBM cells with astrocytes or microglia)	Matrix-free protocol requiring no specialized equipment; precise control over initial cell seeding density and spheroid size; high morphological uniformity	Low experimental throughput (few conditions per place); technical difficulty in media exchange and drug treatment administration; susceptibility to evaporation; challenging spheroid handling and harvesting
Agitation-based culture - Spheroids	Dynamic cell suspension maintained under continuous agitation, increasing cell-to-cell collision frequency and promoting spontaneous self-aggregation; applied for large-scale production of patient-derived cultures	Large scale spheroid production; enhanced nutrient distribution and waste removal; suitable for downstream applications requiring higher quantities of material	High operational cost; substantial media consumption; heterogeneous spheroid size and morphology; shear stress damage to cells; restricted real-time imaging and optical monitoring
Magnetic levitation systems - Spheroids	Cell aggregation mediated by magnetic nanoparticles or contrast agents; applied in GBM to construct spatially controlled co-cultures	Rapid spheroid assembly; capacity to generate heterotypic multicellular structures and co-cultures; precise spatial manipulation of cells; adaptability for screening platforms	Requirement for magnetic nanoparticles/agents and specialized equipment; higher operational costs; potential concentration-dependent effects of particles on cell biology; lack of global standardization across manufacturers and countries
Scaffold-based			
Create an artificial 3D matrix that facilitates cell-ECM interactions, invasion, migration, and mimics tissue-like organization.			
Polymers and hydrogels - Organoids	Generation of a 3D matrix that recapitulates the biochemical and mechanical properties of the native ECM, enabling cell-growth, invasion, migration, and cell-matrix interactions	High biocompatibility with diverse materials; simulates brain ECM composition; tunable mechanical and chemical properties; cell encapsulation capability; suitable for cell-ECM interactions and invasion assays	Batch-to-batch composition variability; failure of certain matrices to fully recapitulate the tumoral ECM; technical difficulty in cell harvesting and downstream processing
Microfluidic systems - GBM-on-a-chip	Controlled microenvironments for studying tumor-microenvironment (TME) interactions; integration of GBM cells with brain microvascular, endothelial and glial cells	Low sample and reagent volume consumption; precise spatiotemporal control; generation of chemical gradients; microvascularization; facilitation of co-cultures; high throughput screening compatibility; real-time monitoring; modeling of complex TME interactions	Complex fabrication and operation; requirement for specialized equipment; limited protocol standardization; challenging integration with conventional analytical assays; low scalability in certain platform designs
3D bioprinting	Deposition-controlled 3D constructs of cells and biomaterials that recapitulate the architectural complexity and heterogeneity of the tumor and its microenvironment; bioink may include GBM cells/GSCs, stromal brain cells, and brain ECM hydrogels to construct complex models	High spatial control; versatility in incorporating multiple cell types and biomaterials; replication of matrix stiffness and chemical gradients; complex multicellular tumor modeling; vascularization potential; personalized drug screening models	High operational cost; limited protocol standardization; bioink selection is critical; printing procedures may compromise cell viability; technical limitations in resolution or viscosity control; scarcity of commercially available bioinks that fully recapitulate the native tumoral ECM

*CSC, cancer stem cell; EMC, extracellular matrix; GSC, glioma stem cell; TME, tumor microenvironment; ULA, ultra-low attachment. References: Abuwatfa et al. (2024), Carvalho et al. (2015), Germain et al. (2022), Jacob et al. (2020), Habanjar et al. (2021), Pinto et al. (2020), Sung and Beebe (2014), and Weiswald et al. (2015).

### 4.1 Cellular models in GBM: characteristics and origin

Among the studies summarized in this review evaluating TMZ resistance in GBM, we identified 143 distinct cellular models, highlighting the diversity of *in vitro* systems used. U-87MG and its derivatives were the most frequently used GBM cell lines for 3D spheroid generation (*n* = 44), followed by patient-derived models, including patient-derived organoids (PDOs, *n* = 23) and patient-derived glioma cells (PDGCs, *n* = 19). Other established cell lines such as U-251MG (*n* = 10) and LN-229 (*n* = 11), T98G (*n* = 6), A-172 (*n* = 5), U-118MG (*n* = 3), and U-373MG (*n* = 2) were less commonly applied. Several additional models were identified only once across the analyzed studies, suggesting substantial heterogeneity and limited standardization in model selection among GBM studies. These cell lines exhibit distinct molecular characteristics, including *TP53* mutation status and *MGMT* promoter hypermethylation profile (Table 2), which can influence various functional mechanisms, particularly TMZ sensitivity.

**Table 2:** GBM cell line models used to evaluate temozolomide resistance in 3D culture.

Cell line	*TP53* status*	MGMT promoter methylation profile*	TMZ sensitivity	References
U-118MG	Mutant (R213Q)	Methylated	Sensitive	Brandt et al. (2023)
LN-229	Mutant (R273H)	Partially Methylated	Sensitive	Tan et al. (2023)
A-172	Wild type	Methylated	Sensitive	Pinevich et al. (2023)
U-251MG	Mutant (R273H)	Methylated	Sensitive	Anan et al. (2024)
U-373MG	Mutant (R273H)	Methylated	Sensitive	Cho et al. (2017) and Anan et al. (2024)
U-87MG	Wild type	Methylated	Moderate resistance	Balça-Silva et al. (2017) and Lee et al. (2020)
T98G	Mutant (M237I)	Non-methylated	Highly resistant	–

*The molecular characteristics and temozolomide (TMZ) sensitivity of the cell lines are described according to information available in cell repositories and public databases, including the ATCC (American Type Culture Collection), ECACC (European Collection of Authenticated Cell Cultures), CCLE (Cancer Cell Line Encyclopedia), and COSMIC, as well as in the specialized literature; (−) no information.

Several GBM cell lines widely used in experimental studies were originally established at Uppsala University, Sweden, and are designated with the prefix “U” (Uppsala) and “MG” (malignant glioma) (Pontén and Macintyre, 1968; Fogh et al., 1977). Among these, U-87MG is one of the most widely used GBM models because of its extensive characterization in the literature. Originally established from a patient diagnosed with glioblastoma/malignant astrocytoma (WHO grade IV), the commercially distributed U-87MG/ATCC line has been reported to be genetically distinct from the original Uppsala line and from the original tumor specimen. Samiei et al. (2020) compared TMZ sensitivity between U-87MG and U-251MG and demonstrated that U-251MG was more sensitive to TMZ in both 2D and 3D models.

Similarly, U-251MG, originally derived from a patient with glioblastoma (WHO grade IV), was historically misidentified as U-373MG due to cross-contamination, and short tandem repeat (STR) profiling later showed that many stocks labeled U-373MG were in fact U-251MG (Torsvik et al., 2014). Other commonly used models include U-118MG, LN-229, T98G, A-172, and U-373MG, each presenting distinct molecular and biological characteristics. For example, T98G has an unmethylated *MGMT* promoter and high *MGMT* expression, contributing to its intrinsic resistance to TMZ. Diao et al. (2019) compared 4 glioblastoma cell lines considering cell morphology, proliferation, migration, and invasion characteristics, and classified them into three distinct types: expansive-growth cells (LN-229 and U-251MG), characterized by high proliferation and limited invasion capacity; individual invasion cells (SNB-19); and cluster invasion cells (U-87MG).

Despite their accessibility, reproducibility, and ease of culture, the use of long-term-established GBM cell lines as representative models of primary tumors has increasingly been questioned. Prolonged adaptation to conventional culture conditions can drive genetic, epigenetic, and phenotypic alterations and may reduce clinically relevant tumor heterogeneity (Boccellato and Rehm, 2022). Consequently, findings obtained from established cell lines, particularly when based on a single model, may not fully reflect the biological complexity or therapeutic response of GBM in patients.

These limitations have contributed to the growing use of GSCs and other patient-derived models, which can better preserve the genotypic, phenotypic, and epigenetic features of the original tumor that may change during prolonged *in vitro* culture (Yashin et al., 2023). Patient-derived GSCs represent a particularly relevant model because they are associated with tumor initiation, therapeutic resistance, and GBM recurrence. Pollard and colleagues successfully isolated and cultured patient-derived GSCs, establishing cultures that preserved tumor-specific characteristics *in vitro* and providing an important model for studying GBM biology (Pollard et al., 2009). GSCs exhibit enhanced DNA damage response capacity, metabolic plasticity, activation of pro-survival signaling pathways, and increased resistance to replication stress, all of which contribute to reduced sensitivity to TMZ (Dirkse et al., 2019). Patient-derived GSCs can be maintained in different 3D configurations, including neurospheres and organoid-based models, which may better preserve clinically relevant features of GBM than long-term-established cell lines. Such patient-derived 3D models are particularly valuable for investigating therapeutic response and chemoresistance, and may better recapitulate clinically relevant aspects of GBM pathophysiology and tumor heterogeneity (Azzarelli, 2020; Pernik et al., 2021; Sha et al., 2025; Park et al., 2026). However, these models still present challenges, including limited sample availability, marked inter-patient variability, elevated culture costs, and technical challenges associated with long-term maintenance and standardization (da Hora et al., 2019). Section 5 discusses the characteristics, nomenclature, and applications of different patient-derived 3D GBM models in detail.

In summary, established GBM cell lines remain important experimental tools due to their reproducibility, accessibility, and ease of handling. However, long-term adaptation to *in vitro* culture conditions and limited preservation of tumor heterogeneity pose significant limitations, particularly in studies focused on therapeutic response and chemoresistance mechanisms. In contrast, this review showed that patient-derived models are among the most commonly employed cell culture systems in studies of therapeutic response in GBM, largely because they better preserve tumor-specific molecular features and clinically relevant resistance patterns. Therefore, careful selection and characterization of cellular models, considering their origin, molecular profile, and biological behavior, are essential for generating robust and clinically meaningful insights into TMZ sensitivity in GBM.

### 4.2 TMZ response in different GBM cells

Across the studies included in this review, TMZ concentrations varied widely among glioblastoma models, ranging from 0.001 to 2000 μM, with substantial differences in treatment duration, experimental design, and therapeutic response. Despite this heterogeneity, some consistent patterns emerged that may help guide the selection of appropriate cellular models based on each study’s objectives.

U-251MG spheroids were predominantly treated with intermediate TMZ concentrations of 100, 200, and 500 μM, although lower concentrations of 25 and 50 μM were also reported (Ribeiro et al., 2024). Across these treatment conditions, U-251MG spheroids generally showed limited reductions in cell viability and spheroid growth, indicating a relatively weak response to TMZ in 3D culture. This finding contrasts with the TMZ-sensitive phenotype commonly observed in U-251MG cells in conventional 2D cultures. Thus, the increased resistance observed in 3D models suggests that microenvironmental and architectural features of spheroid culture may substantially influence the therapeutic response to TMZ.

In contrast, T98G spheroids were exposed to higher TMZ concentrations, including 600, 1,000, and 2000 μM. Notably, the IC₅₀ reported for this cell line may reach 1351.44 μM, as reported by Lee et al. (2024). This pronounced resistance may reflect its molecular profile, particularly high MGMT expression and enhanced DNA damage repair capacity, both of which can counteract TMZ-induced cytotoxicity. Additionally, T98G cells exhibit increased tolerance to replication stress and cell cycle checkpoint activation, further contributing to their resistant phenotype.

Intermediate TMZ concentrations, around 100 μM or within the range of 100–500 μM, were most frequently employed in U-87MG (Nakod et al., 2022; Yadav et al., 2022; Pérez-Aliacar et al., 2024) and LN-229 (Singh et al., 2020; Phour and Vassella, 2024; Sun et al., 2025) spheroids. In these models, reductions in cell viability and spheroid size were observed more consistently, suggesting greater sensitivity to TMZ. Importantly, these models are also commonly used to investigate mechanistic aspects of DNA damage response signaling, stemness-associated pathways, and therapeutic resistance.

Another important group comprises patient-derived models, PDOs and PDGCs, which may better preserve tumor heterogeneity and clinically relevant molecular features than established cell lines. Across these models, substantial variability was observed in both the TMZ concentrations required to elicit a response and the magnitude of treatment effects. Some patient-derived models showed moderate sensitivity to TMZ, with reduced cell viability and disruption of 3D architecture at concentrations around 100 μM (Majc et al., 2024; Phour and Vassella, 2024; Díaz Méndez et al., 2025; Khotchawan et al., 2025; Potthoff et al., 2025), others remained resistant even at concentrations of 150–200 μM or higher (Abbas et al., 2020; Avci et al., 2020; Hwang et al., 2020; Park et al., 2021; Gozdz et al., 2022; Morelli et al., 2022; Rodrigues-Junior et al., 2022; Qu et al., 2023; Oraiopoulou et al., 2024; Chuang et al., 2025; Hincapié-Arias et al., 2025; Na et al., 2025; Gao et al., 2026).

This variability has been associated with several molecular determinants, particularly MGMT promoter methylation status, DNA damage response capacity, stemness-associated programs, and microenvironmental adaptation, as reported by Verduin et al. (2023) and Potthoff et al. (2025). Moreover, GSC-containing patient-derived models frequently exhibit hypoxic gradients, ECM interactions, and increased intratumoral heterogeneity, all of which may directly influence TMZ penetration and therapeutic response.

Overall, these findings highlight that TMZ efficacy in 3D GBM models is not solely dose-dependent but is also strongly influenced by the cellular model, its molecular background, and the complexity of the 3D system microenvironment. While established cell lines are valuable for mechanistic studies, patient-derived models, particularly those that preserve GSC populations, may provide responses that more closely resemble clinically relevant, patient-specific patterns of therapeutic resistance. Importantly, the absence of a consistent dose–response relationship across different GBM models underscores the need for more physiologically relevant experimental systems and therapeutic strategies to overcome TMZ resistance mechanisms. Furthermore, the use of supraphysiological TMZ concentrations in several studies underscores the need to incorporate clinically relevant drug-exposure conditions into future 3D GBM models.

## 5 Three-dimensional culture models of glioblastoma

### 5.1 Establishment of three-dimensional cultures

3D cell culture allows cells to self-organize into concentric layers that more closely mimic tumor architecture and physiology. In general, 3D cultures can be classified as scaffold-based or scaffold-free (Sakalem et al., 2021) (Figure 3). In scaffold-free systems, cells self-assemble into tissue-like structures without an external matrix, generating free-floating 3D multicellular aggregates that provide versatile experimental configurations (Abuwatfa et al., 2024; Sreepadmanabh et al., 2024). These models include (a) hanging drop, (b) ultra-low adhesion (ULA) plates, (c) agitation-based culture, (d) magnetic levitation systems, and (e) tumorspheres (Liu et al., 2024) (Figures 3a–e).

**Figure 3:**
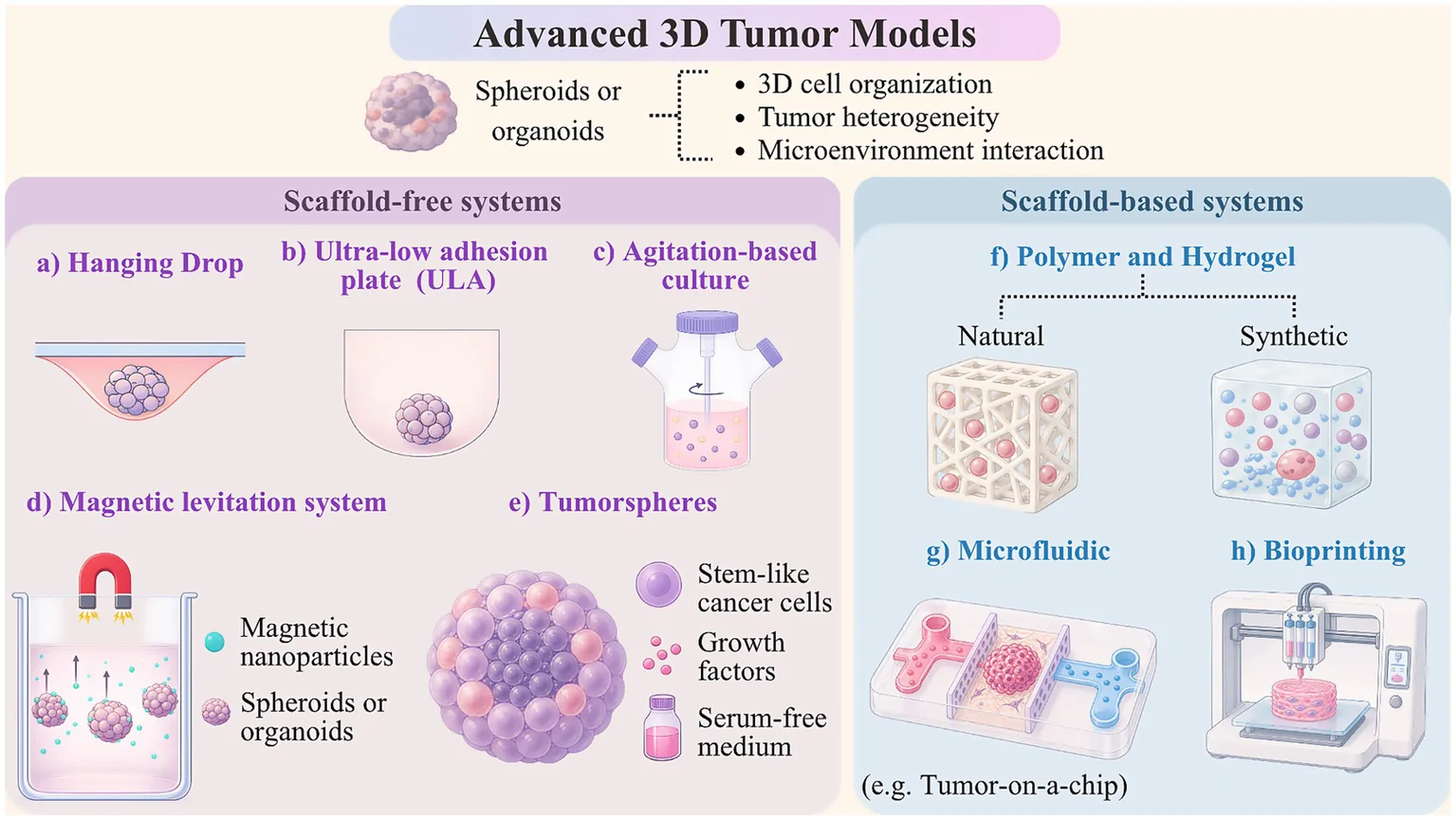
The main available 3D cell culture technologies are divided into scaffold-free and scaffold-based. **(a)** Hanging Drop. **(b)** Ultra-low adhesion plate (ULA). **(c)** Agitation-based culture. **(d)** Magnetic levitation system. **(e)** Tumorspheres. **(f)** Polymer and Hydrogel. **(g)** Microfluidic. **(h)** Bioprinting.

Some cell lines cannot self-aggregate and therefore require a “scaffold” to form stable 3D structures. Moreover, several cell types depend on substrate adhesion for survival and, in its absence, undergo anoikis-mediated cell death (Sreepadmanabh et al., 2024). To address these limitations, researchers have explored scaffold-based techniques. Scaffolds are porous, fibrous, or permeable biomaterials that support transport of body fluids and gases and promote cell interactions, viability, and ECM formation without inducing toxicity or an inflammatory response (Nikolova and Chavali, 2019). These systems include polymer scaffolds, hydrogels (natural or synthetic), decellularized tissue scaffolds, and hybrid scaffolds that incorporate microfluidic devices (Habanjar et al., 2021) (Figures 3f–h).

### 5.2 Spheroid vs. organoid

Cell cultures have substantially reduced the use of animal models in biomedical research; however, conventional 2D monolayer cultures fail to adequately recapitulate the tissue architecture, cellular heterogeneity, and tumor microenvironmental dynamics, leading to altered cellular responses and reducing their effectiveness for preclinical drug screening (Jensen and Teng, 2020; Sakalem et al., 2021). Although they provide a useful starting point for exploring brain tumor biology and responses to therapies such as TMZ, conventional 2D cell cultures fail to reproduce the cellular heterogeneity, cell-to-cell and cell-to-microenvironment interactions, and tissue complexity of glioma/glioblastoma. Therefore, the complexity of the GBM ecosystem requires the use and continued development of more physiologically relevant models, including 3D cultures, capable of more accurately recapitulating tumor initiation, progression, invasion, cellular heterogeneity, and therapeutic response (Robertson et al., 2019; Azzarelli, 2020; Raju et al., 2024). Table 3 summarizes the main advantages and limitations of 2D cultures, 3D models, and *in vivo* systems for investigating TMZ response in GBM.

**Table 3:** Comparative advantages and limitations of 2D cultures, 3D models, and *in vivo* models for assessing TMZ response in glioblastoma.

Model	Advantages	Limitations	Relevance for TMZ studies
2D cultures 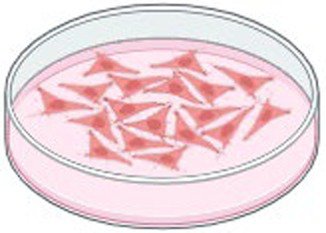	Low-cost and highly reproducible, with easy manipulation and applications in large scale screening and mechanistic studies	Limited cell–cell/ECM interactions; absence of spatial gradients; altered morphology and drug exposure	Provides a useful model for initial screening and characterization of intrinsic TMZ responses; however, drug sensitivity may be overestimated in this model
3D cultures 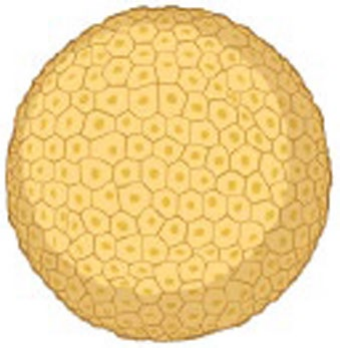	Better preserves tumor architecture, cell–cell and cell–ECM interactions, cellular heterogeneity, oxygen and nutrient gradients, and barriers to drug penetration	Greater variability; higher cost and technical complexity; limited standardization across platforms	More closely reflects microenvironment-dependent TMZ responses and resistance mechanisms and enables the assessment of drug penetration and heterogeneous responses
*In vivo* models 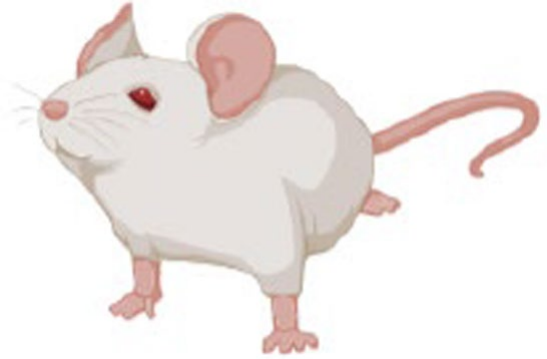	Highest physiological complexity; systemic pharmacokinetics; tumor–host interactions and vascularization	High cost; low throughput; ethical considerations; species-specific differences; limited experimental accessibility	Provides important information on systemic exposure, toxicity, pharmacokinetics, and therapeutic efficacy, but has limitations for controlled mechanistic studies

The illustrations were created using the free version of BioRender (BioRender.com).

The terms “organoids,” “spheroids,” and “3D cell cultures” are frequently used interchangeably in the literature, although these systems differ substantially in biological complexity and cellular origin. Spheroids, also called multicellular tumor spheroids (MCTS), are typically generated from established cancer cell lines or, less frequently, from tumor tissue fragments. Although they show limited histological resemblance to the original tumor, spheroids partially reproduce several *in vivo* tumor features, including metabolic gradients, proliferation dynamics, hypoxia, and nutrient diffusion limitations (Gunti et al., 2021).

In general, spheroids are composed of three concentric cellular layers, arranged from the center toward the periphery: (i) a necrotic core; (ii) a quiescent zone containing viable but non-proliferative cells; and (iii) a proliferative layer composed of actively dividing cells (Figure 4). Other terms related to 3D cell cultures can be further described. Assembloids are modular models that enable the study of cells and brain regions, either isolated or integrated to other organoids (Miura et al., 2022). Neurospheres are spheroids that assemble *in vitro* when mitotic CNS cells are plated on a nonadhesive substrate (Singec et al., 2006). Tumor spheroids are spheroids derived from cancer stem cells and correspond to 3D cultures of individual tumor stem cells under serum-free conditions. Similarly, some authors use the term “tumorspheres” (Vargas-Toscano et al., 2020; Park et al., 2021; Majc et al., 2024; Sørensen et al., 2024; Díaz Méndez et al., 2025; Na et al., 2025; Tehrani et al., 2025) to refer to neurospheres in the context of gliomas. These models are scaffold-free 3D cultures generated by clonal expansion of tumor cells maintained in suspension, whether derived from tumor tissues, circulating tumor cells, or established cell lines (da Silva Siqueira et al., 2021). This 3D tumor model can maintain cancer stem cell (CSC) characteristics and reproduce cell–cell interactions, as well as oxygen and nutrient gradients. These stem-like cell populations express several stemness-associated markers, including B cell-specific Monoley murine leukemia virus integration site 1 (BMI1), SRY-box transcription factor 2 (SOX2), Neuroepithelial stem cell protein (NESTIN), Oligodendrocyte transcription factor 2 (OLIG2), Nanog homeobox (NANOG), MYC proto-oncogene (MYC), and Inhibitor of DNA binding 1 (ID1), and exhibit enhanced tumor-relevant features, such as self-renewal capacity and therapeutic resistance (Ruiz-Garcia et al., 2020). Tumor cell aggregates are multicellular tumor spheroids generated from one or more established cell lines, and these models exhibit a 3D structure that resembles micro-tumors or metastases (Dubessy et al., 2000; Kingsley et al., 2019). These 3D cultures can be cultivated using a substrate or scaffold, or be scaffold-free (Sakalem et al., 2021). The following topics cover the subtypes of 3D models that can be used to investigate GBM.

**Figure 4:**
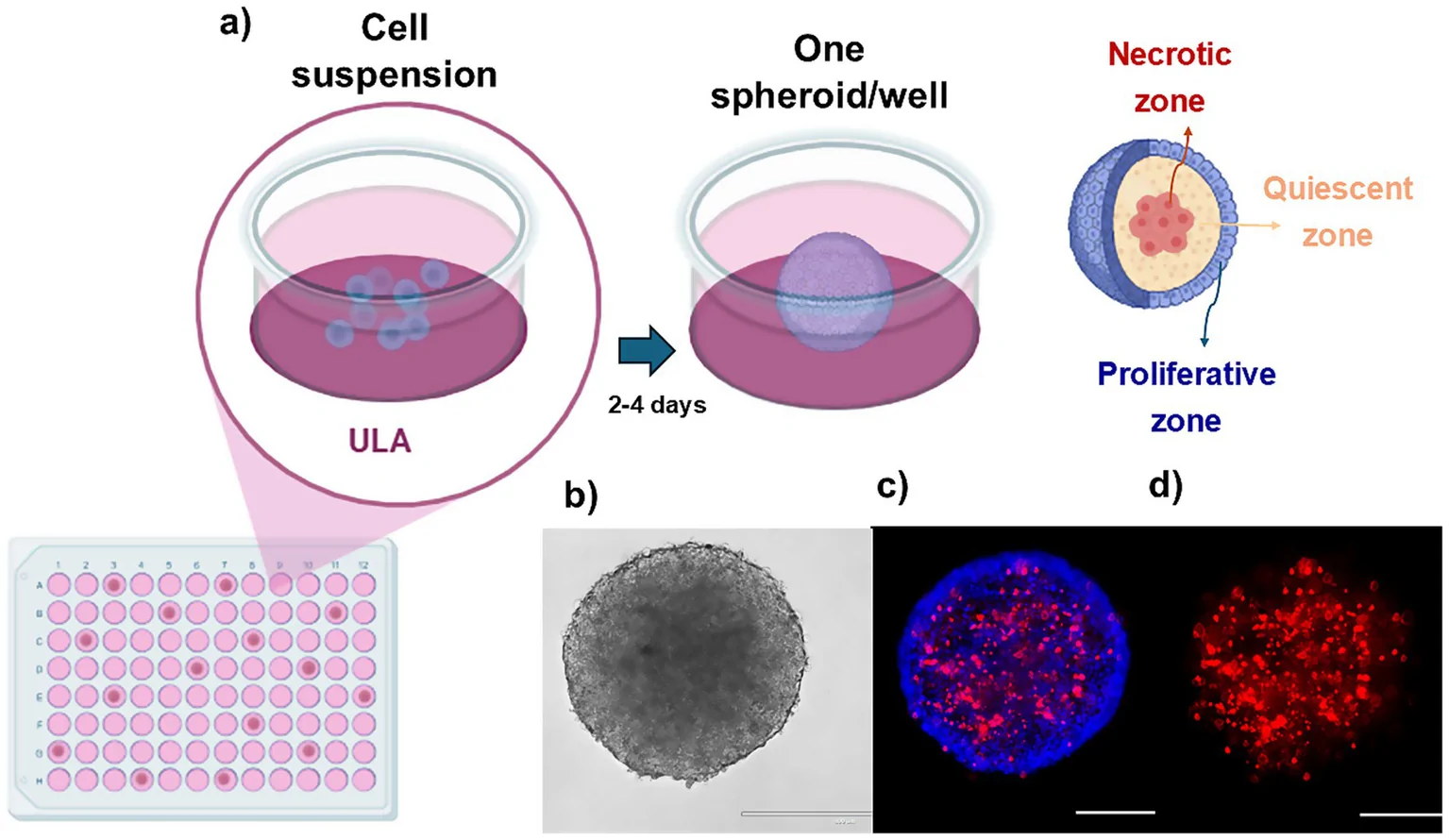
**(a)** Schematic illustration of the glioblastoma spheroid generation protocol using ultra-low-attachment round-bottom 96-well plates (purple). **(b)** A schematic diagram illustrating the internal structure of the spheroids is provided together with bright-field and immunofluorescence images of the spheroid; live cells exhibit intense blue fluorescence of nuclei (Hoechst), and dead cells exhibit red fluorescence (propidium iodide). The scale bars represent 200 μm. Images were captured using an Evos-FL fluorescence microscope with a 20X objective.

#### 5.2.1 Brain tumor organoid models

Spheroids are valuable tools for studying basic cellular processes, drug screening, and tumor biology. However, organoids are more complex structures that can partially recapitulate tissue architecture, multicellular interactions, and organ-level physiology (Živković and Opačak-Bernardi, 2025). Importantly, organoids better preserve tumor heterogeneity and microenvironmental complexity than conventional spheroid models. Hubert et al. (2016) first generated GMB organoids. They demonstrated that their cellular and microenvironmental heterogeneity enabled the simultaneous maintenance of functionally and phenotypically distinct glioblastoma stem-like and non-stem cell populations.

Researchers have developed several methodologies to generate brain tumor organoids, which differ in technical complexity, cellular source, and biological relevance. These approaches include: (i) PDOs, including organoids established from patient-derived tumor tissue or cells, which can retain important histological, cellular, molecular, and genetic features of the parental tumor (Jacob et al., 2020); (ii) organoid models of brain tumors, including brain or cerebral organoids “mini-brains,” which may or may not be genetically engineered through the introduction of oncogenic alterations into ESC- or iPSC-derived neural/brain organoids to model tumor initiation and progression (Bian et al., 2018); and (iii) brain tumor assembloids, which combine tumor cells, tumor-derived organoids or spheroids, and/or tumor tissue with healthy brain organoids or other neural cell populations to model interactions between tumor cells and the brain microenvironment. Within this category, cerebral organoid glioma (GLICO) models represent a specific approach in which patient-derived GSCs are co-cultured with human cerebral organoids, enabling study of tumor invasion and interactions with surrounding neural tissue (Linkous et al., 2019). Other tumor–brain assembloid approaches have subsequently combined cerebral organoids with tumor tissue or tumor spheroids to generate more complex models of the tumor microenvironment (Heinzelmann et al., 2024; de Lucas Sanz et al., 2026).

Among these approaches, PDOs are currently the most widely employed because they better preserve the genetic, epigenetic, histopathological, and phenotypic heterogeneity of the original tumor (Zhou et al., 2025). These 3D structures, also known as “tumoroids,” comprise multiple tumor-associated cell populations and partially reproduce key features of the parental tumor (Soubéran et al., 2025). In most cases, PDO generation does not require ECM supplementation because viable tumor pieces and dissociated cell suspensions can spontaneously self-assemble into 3D structures (de Lucas Sanz et al., 2026). Given GBM’s inter- and intratumoral heterogeneity, including distinct molecular subtypes, patient-derived models are particularly valuable for investigating TMZ responsiveness and developing precision medicine approaches.

#### 5.2.2 Patient-derived models for TMZ response and resistance

This review highlights patient-derived models as essential tools for studying TMZ response and resistance in GBM. These models include PDOs (*n* = 23) and PDGCs (*n* = 19), which differ in their complexity. PDOs are three-dimensional models derived from patient tumor tissue that can preserve intratumoral heterogeneity and tumor–microenvironment interactions. This makes them especially useful for studying tumor biology, therapeutic responses, and resistance mechanisms. In contrast, PDGCs are directly isolated from patients and cultured as primary cells, such as adherent cultures, neurospheres, and other 3D structures. PDGCs are generally easier and less expensive to establish, allowing for more flexible experiments, though their *in vitro* adaptation can lead to loss of some original tumor characteristics (Klein et al., 2020). Although PDOs are more technically complex, they are increasingly used for drug screening and assessing therapeutic responses, supporting their role in preclinical drug development (Hou et al., 2022). Nonetheless, PDO creation is time-consuming, costly, and technically demanding, making PDGCs a more accessible and manageable alternative. Overall, PDOs and PDGCs offer complementary methods for exploring patient-specific tumor biology and treatment responses, differing mainly in structural complexity and ease of use.

Research using patient-derived models has examined various molecular factors and therapeutic mechanisms related to TMZ response and resistance. Several studies have focused on MGMT promoter methylation’s role, while others have explored resistance pathways such as activation of the epidermal growth factor receptor (ErbB) signaling pathway (Fabro et al., 2022), increased Collagen Type VI Alpha 1 Chain (*COL6A1*) expression (Gao et al., 2026), and improved responses when combining TMZ with NF-κB pathway inhibitors (Avci et al., 2020). Recent research has expanded PDO applications to study molecular determinants of TMZ response and explore treatment strategies (Park et al., 2026; Sha et al., 2025). PDGC-based studies, especially those using patient-derived glioma stem-like cell cultures and 3D sphere models, have also advanced understanding of stemness, treatment resistance, and personalized drug responses (Park et al., 2021; Avenel et al., 2024; Na et al., 2025). Overall, these studies show how patient-derived models can complement traditional GBM cell lines by reflecting clinically relevant tumor heterogeneity and offering diverse biological and structural modeling systems. Supplementary Table 1 summarizes additional applications of patient-derived models in TMZ chemoresistance studies.

Established brain organoids from ESCs that recapitulate three-dimensional neural organization, using a protocol involving embryoid body formation, neural induction, matrigel embedding, and tissue expansion (Lancaster et al., 2013). Organoids can also be developed from induced pluripotent stem cells (iPSCs), providing a versatile platform for modeling glioblastoma cells and therapeutic response (Xu et al., 2023). These brain organoids closely mimic human brain organization and have become valuable experimental systems for studying neurological diseases (Wen et al., 2023).

In this review, two studies used iPSC-derived systems to evaluate TMZ effects in glioblastoma organoids. Hwang et al. (2020) generated an iPSC line from bone marrow-derived mononuclear cells obtained from a patient harboring a c-MET mutation, a molecular alteration previously associated with aggressive GBM phenotypes (Anton et al., 2021). Using ultra-low attachment (ULA) plates, the authors generated iPSC A-derived neuronal-like organoids that, after 90 days of differentiation, exhibited GBM-like characteristics. TMZ treatment increased caspase-3 activation, suggesting that these organoids may represent useful platforms for evaluating TMZ-induced toxicity (Hwang et al., 2020).

Peng et al. (2025) developed three distinct 3D culture systems: individualized patient tumor organoids (IPTOs), PDOs, and the Glioma/Glioblastoma Cerebral Organoid (GLICO) method. The authors showed that IPTOs are the most effective model for maintaining tumor microenvironment components and preserving patient-specific histopathological characteristics. In the GLICO model, iPSC-derived organoids were co-cultured with patient-derived GBM tumor cells, while in IPTOs, the tumor fragments were directly inserted into the cerebral organoid. TMZ sensitivity assessed in IPTOs correlated with patient clinical outcomes, as TMZ-sensitive IPTOs were associated with improved progression-free and overall survival compared with TMZ-resistant IPTOs (Peng et al., 2025).

Russo et al. (2026) also used the GLICO model with human embryonic stem cell-derived cerebral organoids containing GBM tumor cells, and TMZ (100 μM) decreased spheroid growth after 7 days of treatment. The GLICO model established by Linkous et al. (2019) closely phenocopies glioblastoma invasion dynamics, as tumor microtubes facilitate extensive infiltration into the host neural tissue.

Regarding assembloids, only one study in the present literature review investigated TMZ resistance. Sun et al. (2025) generated cerebral organoids from iPSCs (DYR0100) and co-cultured them with different GBM cells (*HSD52* knockout vs. control conditions). The authors identified the noncoding RNA HSD52 as significantly upregulated in TMZ-resistant GBM organoids and demonstrated that this molecule promotes resistance through multiple mechanisms, including DNA hypomethylation-mediated activation of the ATM signaling pathway.

From a bioengineering perspective, organoid systems can be generated using conventional scaffold-free approaches, integrated into microfluidic platforms (organoid-on-a-chip), or fabricated through 3D bioprinting technologies (Li Z. et al., 2025). These emerging technologies may further improve reproducibility, microenvironmental control, vascularization, and translational applicability of GBM organoid systems.

### 5.3 Scaffold-free 3D cultures

The scaffold-free approach, based on the spontaneous self-assembly of cells into multicellular spheroids, represents one of the most widely used strategies for 3D tumor culture (Manikandan and Jaiswal, 2023). In the present investigation, articles evaluated the TMZ response in scaffold-free spheroids using ultra-low attachment (ULA) plates (*n* = 49), tumorsphere (*n* = 7), hanging-drop (*n* = 8), agitation-based culture (*n* = 1), and magnetic levitation systems (*n* = 1).

ULA plates are culture plates coated with a hydrophilic polymer, most often agarose, that prevents protein adsorption and cell adhesion onto the culture flask surface (Wanigasekara et al., 2025). This method is rapid, reproducible, and cost-effective for screening different substances, concentrations, and treatment combinations. However, not all GBM cell lines commonly used in 2D studies form spheroids efficiently under 3D culture conditions. For instance, Bach et al. (2024) tested ten GBM cell lines (1,321 N1, U-251MG, U-87, T98G, LN-229, MZ-54, G55T2, MZ-18, LN-405, and U-343MG) and reported that spheroids generated from the LN-405, MZ-18, and MZ-54 cell lines failed to sustain growth.

Using the ULA technique, Phour and Vassella (2024) demonstrated that LN-229 cells form perfectly spherical, compact structures, whereas LN-18 cells form irregular, non-uniform aggregates; however, the authors did not explore the mechanisms underlying these morphological differences in spheroid conformation. Round, agglomerated spheroids form with very strong cell–cell adhesion, whereas grape-like and stellate conformations result from poor cell–cell adhesion (Parkar et al., 2024).

Tumorsphere formation depends on specific culture conditions that maintain cell growth in low-adhesion environments while preserving an undifferentiated phenotype. In these conditions, defined culture media such as DMEM/F12 (Na et al., 2025) or Neurobasal medium (Majc et al., 2024; Sørensen et al., 2024; Tehrani et al., 2025) are commonly used without fetal bovine serum (FBS) to prevent cellular differentiation. These media are supplemented with additives such as B27 (without vitamin A) and N2, which provide metabolic and nutritional support while maintaining stem-like properties (Park et al., 2021; Majc et al., 2024; Sørensen et al., 2024; Díaz Méndez et al., 2025; Na et al., 2025; Tehrani et al., 2025). In addition, growth factors such as Epidermal Growth Factor (EGF) and basic Fibroblast Growth Factor (bFGF/FGF-2) are included because they support cell proliferation and self-renewal. Other frequently used supplements include heparin and L-glutamine, which support cellular metabolism and pH stability.

The hanging drop technique is one of the most labor-intensive scaffold-free 3D approaches. In this system, droplets containing cell suspensions are dispensed onto the underside of a culture plate lid, where they remain suspended due to surface tension. After a few hours, gravity promotes spontaneous cell aggregation and spheroid formation (Nath and Devi, 2016). In this review, U-251MG, U-87MG, U-118MG, T98, and LN-229 cell lines formed spheroids using this technique, and four studies employed the hanging-drop technique to generate patient-derived models (Morelli et al., 2022; Lam et al., 2023; Oraiopoulou et al., 2024; Hincapié-Arias et al., 2025).

Furthermore, among the included studies, only one used agitation-based culture (Potthoff et al., 2025), while another used magnetic levitation systems (Datta et al., 2024). Agitation-based cultures promote spontaneous cell aggregation into spheroids through continuous movement, typically achieved using orbital shakers or rotating flasks, thereby preventing cell adhesion to the culture vessel (Santo et al., 2016). This 3D culture method offers advantages, including improved nutrient and oxygen distribution and enhanced cell–cell interactions (Franchi-Mendes et al., 2021). In contrast, magnetic levitation systems incorporate magnetic nanoparticles into cells, which an external magnetic field then guides to induce spheroid formation, providing a more controlled and rapid environment for cell aggregation (Tepe et al., 2023). Although less commonly used than conventional methods, these approaches offer alternative scaffold-free strategies for generating tumor spheroids.

### 5.4 Scaffold-based 3D cultures

Scaffold-based systems have been widely employed to study complex interactions between cells and the tumor microenvironment, serving as valuable models for culturing patient-derived tumor cells, screening therapeutic drugs, and evaluating treatment efficacy (Unnikrishnan et al., 2021). Studies in this review evaluating TMZ activity in GBM cells used hydrogels (*n* = 28), microfluidic systems (*n* = 8), and bioprinting approaches (*n* = 6).

#### 5.4.1 Polymer and hydrogel

Polymers are macromolecules composed of repeating structural units that can be either naturally derived or synthetically produced (Liu and Wang, 2020). In 3D cell culture systems, polymers are widely used as scaffold-based materials to support cell growth and organization, and they may exist as either hydrogels or more solid structures. Although many studies use polymers to form hydrogels, several polymers do not form hydrogels and instead serve as structural supports in the form of fibers, films, or porous scaffolds (Perez-Puyana et al., 2020; Parkar et al., 2024). Examples of non-hydrogel-forming polymers include polylactic acid (PLA), polyglycolic acid (PGA), and polycaprolactone (PCL) (Brown et al., 2023; Liu et al., 2024). These materials commonly provide rigid or semi-rigid frameworks that influence cell adhesion, morphology, and mechanical stability (Fan et al., 2019). However, none of these polymers was specifically employed in the TMZ studies included in this review. Although these polymers can support 3D cell growth, hydrogel-forming materials more effectively reproduce extracellular matrix (ECM) characteristics.

Hydrogel scaffolds are a subset of polymer-based scaffolds that absorb and retain large amounts of water, forming a gel-like matrix (Bittman-Soto et al., 2024). These biomaterials consist of crosslinked hydrophilic polymers immersed in liquid culture medium (Sreepadmanabh et al., 2024). Hydrogels can be classified as natural or synthetic. Natural hydrogels, such as proteins and polysaccharides derived from biological sources, generally exhibit excellent biocompatibility and low toxicity (Ruedinger et al., 2015). The most common are collagen, collagen-chitosan, matrigel, fibrinogen, alginate, albumin, agarose, gelatin, hyaluronic acid, among others (Edmondson et al., 2014; Thai et al., 2026). Synthetic hydrogels offer a well-defined chemical structure and improved reproducibility. Common examples include poly(ethylene glycol) (PEG) and polyethylene glycol diacrylate (PEGDA) (Ruedinger et al., 2015; Parkar et al., 2024).

Matrigel is the most widely used natural hydrogel and is often considered the gold standard basement membrane extract. Both Matrigel and Cultrex consist primarily of laminin, type IV collagen, entactin, proteoglycans, and growth factors (Kleinman and Martin, 2005; Prince et al., 2022). Matrigel also appeared most frequently among the articles reviewed in this study (*n* = 11). In contrast, Verduin et al. (2023) used Cultrex only to generate PDOs and compare TMZ response between *MGMT*-methylated and non-methylated samples.

Both methodologies were mainly used in PDO cultures, highlighting the importance of basement membrane components as supportive scaffolds for patient-derived models. Their complex composition may support cell survival, self-organization, and three-dimensional growth while preserving tumor-like features *in vitro* (Cheng et al., 2024). These characteristics are particularly relevant for PDOs, which aim to maintain intratumoral heterogeneity and reproduce patient-specific therapeutic responses (Wolff and Hendrix, 2025). Therefore, these culture systems may represent valuable tools for understanding TMZ sensitivity and resistance.

#### 5.4.2 Microfluids and bioprinting

Among the most advanced 3D culture platforms currently employed in GMB research are microfluidic systems, including tumor-on-a-chip devices, also known as “lab-on-a-chip” (Parkar et al., 2024). These platforms enable controlled fluid flow within microscale channels, facilitating 3D culture formation and recapitulating key aspects of the tumor microenvironment. Such systems have proven particularly valuable for assessing chemotherapeutic efficacy in brain tumor models (Habanjar et al., 2021). In the direct co-culture model, cells placed on the same interface can communicate through direct cell–cell contact, as well as autocrine and paracrine signaling pathways. By contrast, in the indirect co-culture system, cells at separate interfaces do not physically interact and communicate through autocrine and paracrine mechanisms (Li et al., 2024).

In this review, 7 studies used microfluidic approaches to evaluate TMZ effects in glioblastoma models. Samiei et al. (2020) created a glioblastoma-on-a-chip (GoC) model with a microfluidic device containing multiple compartments representing tumor tissue (U-251MG and U-87MG spheroids embedded in a hydrogel scaffold), stromal components, and a vascular structure. While TMZ at 50 μM decreased viability in 2D culture, the GoC model showed a significant reduction only at 100 μM, with U-251MG cells showing greater sensitivity to TMZ than U-87MG cells. Shojaei et al. (2020) similarly demonstrated that TMZ (100 μM) induced apoptosis and cleaved PARP levels in U-251MG and U-87MG spheroids.

Seyfoori et al. (2025) used another microfluidic platform to co-culture U-251MG spheroids embedded in reelin-conditioned ECM and collagen matrices; U-251MG spheroids showed decreased viability, more pronounced in reelin-conditioned ECM at 50 μM, and were less sensitive to TMZ than spheroids in the hydrogel scaffold. Clancy et al. (2022) coupled a microfluidic device with microchannels containing different TMZ concentrations with U-87-MG spheroids (PEGDA and 4-arm PEG-Ac hydrogel scaffold). They showed that cells were less susceptible to TMZ when encapsulated in the degradable, adhesive 4-arm PEG-Ac hydrogel. Microfluidics has also shown that TMZ decreases cell viability in cryopreserved and non-cryopreserved spheroids (Glia et al., 2025). In PDGCs, TMZ increases the expression of tumor progression-associated proteins after co-culture with endothelial cells (Lam et al., 2023) and increases the activity of c-Jun N-terminal kinases (JNKs), which may function as an alternative survival pathway (Fabro et al., 2022).

Bioprinting technologies employ layer-by-layer deposition of bioinks composed of living cells, biomaterials, and biologically active factors to construct tissue-like structures with defined spatial architecture (Piotrowska et al., 2025). This technique can fabricate organs by arranging multiple cell types to closely resemble native tissues (Parkar et al., 2024). Researchers can fabricate 3D bioprinting scaffolds using inkjet-based, laser-assisted, and extrusion-based methods, each with specific strengths, weaknesses, and limitations (Zhang et al., 2021).

Using a pneumatic extrusion system (Inkredible™), Oliver et al. (2024) printed a scaffold with a bioink composed of non-animal-derived polysaccharide components, including alginate and highly hydrated cellulose nanofibrils, PDGCs, and mesenchymal stem cells (MSCs). The authors showed that co-culture with MSCs protected tumor cells from the cytotoxic effects of TMZ and that TMZ downregulated *TREM-1* (Triggering receptor expressed on myeloid cells 1), an immunoglobulin associated with higher GBM grade and poorer outcomes. Pun et al. (2024) combined extrusion bioprinting and stereolithography in a system called GlioFlow3D, using primary human cells and the U-87 cell line in hydrogel-based microchannels that mimic vasculature, and showed that cells in the perivascular region were more resistant to death after TMZ treatment. Bioprinting has also been used to demonstrate TMZ effects in spheroid areas (Han et al., 2020), cell viability in U-87MG cells (Cui et al., 2025; Sambamoorthy et al., 2026), and greater TMZ chemoresistance in T98 cells than in U-87MG cells (Lee et al., 2024).

### 5.5 Tumor microenvironment and its impact on TMZ response in 3D GBM models

Studying the GBM tumor microenvironment (TME) is fundamental, since interactions between tumor cells and stromal components directly influence tumor progression, invasiveness, and therapeutic response. Several studies have demonstrated that 3D models more accurately recapitulate GBM resistance to TMZ than conventional 2D cultures. Using a 3D microfluidic GBM model, Samiei et al. (2020) observed reduced tumor sensitivity to TMZ associated with decreased apoptosis and simultaneous modulation of autophagy and cellular invasion. Supporting these findings, Dragoj et al. (2021) showed that long-term 3D alginate microfiber models containing U-87MG cells exhibited increased expression of chemoresistance-related genes, including *MGMT* and ATP-binding cassette sub-family B member 1 *(ABCB1)*, after TMZ exposure, an effect more pronounced than in monolayer cultures. Similarly, therapeutic resistance in GBM depends not only on interpatient heterogeneity but also on 3D architecture, as PDOs showed differential modulation of kinase pathways, including ErbB signaling, and invasive phenotypes (Fabro et al., 2022).

The TME’s influence on chemoresistance has also been linked to ECM mechanical properties. In evaluating hydrogel-encapsulated spheroids, Hill et al. (2021) showed that degradable and adhesive hydrogels exhibited greater tumor resistance than less permissive systems. Similarly, Bruns et al. (2023) showed that increased microenvironmental stiffness promotes tumor infiltration, hypoxia, and Hypoxia-Inducible Factor 1-Alpha *(HIF-1α)* upregulation, indicating that TME biomechanical properties contribute to more aggressive and therapy-resistant phenotypes.

Beyond biomechanical properties, the spatial organization of the TME and perivascular interactions also play a central role in GBM therapeutic resistance. 3D models incorporating the blood–brain barrier have demonstrated that increasing architectural complexity progressively reduces TMZ sensitivity, suggesting that this barrier itself contributes to the establishment of resistant niches (Lam et al., 2023). Likewise, vascularized bioprinted models containing fibroblasts, endothelial cells, and GBM spheroids showed that vascular structures promote tumor growth and alter TMZ response (Han et al., 2020). Cui et al. (2025) demonstrated that nanoengineered systems, namely extracellular vesicles derived from *Citrus limon*, can serve as biomimetic TMZ nanocarriers that cross endothelial barriers, reduce oxidative stress, and decrease Vascular Endothelial Growth Factor A (VEGF-A) secretion, thereby modulating drug delivery. These naturally occurring lipid-membrane vesicles can encapsulate therapeutic compounds and may help improve drug stability and transport across endothelial barriers. In their results, Cui and colleagues showed that TMZ-loaded citrus-derived vesicles crossed the endothelial barrier while preserving endothelial integrity in their glioblastoma model, enhancing drug delivery and modulating the tumor microenvironment.

PDOs have emerged as one of the most robust platforms for reproducing tumor heterogeneity. These models exhibit spatially organized proliferative niches, heterogeneous tumor stem-cell populations, and reduced responses to combined TMZ and radiotherapy, closely mimicking resistance patterns observed in patients (Sundar et al., 2022). Thus, patient-derived glioblastoma models may provide a more physiologically relevant approach for investigating tumor behavior and assessing therapeutic responses. Linkous and colleagues created the GLICO model by co-culturing patient-derived GSCs with human cerebral organoids (Linkous et al., 2019). In this setup, GSCs migrated toward and invaded the organoid tissue, proliferated inside the host parenchyma, and formed tumors that exhibited several characteristics of the patients’ GBMs. Verduin et al. (2023) demonstrated that PDOs preserve genetic variants, cellular states, and phenotypic heterogeneity while exhibiting differential TMZ responses associated with *MGMT* promoter methylation status. Morelli et al. (2022) further investigated the translational applicability of these systems, using fluorescence lifetime imaging microscopy (FLIM) to perform early metabolic stratification of TMZ response in resected tumor-derived organoids and identify responder and non-responder groups within days after surgery. GBM organoids have also shown TMZ resistance associated with transcriptomic modulation of the Rho GTPase and NOTCH signaling pathways (Morelli et al., 2024).

Studies using patient-derived models and spheroids have also shown that 3D models preserve tumor heterogeneity, maintaining genomic, epigenomic, and transcriptomic features comparable to the primary tumor and displaying dynamic alterations in cellular composition following TMZ exposure (Moore et al., 2026). Watanabe et al. (2024) showed that GBM stem cell-derived organoids exhibit intrinsic immune molecular programs enriched in genes encoding cytokines, interferons, and antigen-processing factors, even without infiltrating immune cells, suggesting that immunoregulatory mechanisms may emerge intrinsically from the 3D architecture. In this context, Special AT-rich sequence-binding protein 2 (SATB2+) and Homeodomain-Only Protein Homeobox (HOPX+) cell populations showed marked resistance to TMZ and radiotherapy, underscoring the importance of cellular heterogeneity in tumor persistence.

3D co-culture models containing patient-derived cells and mesenchymal stem cells differentiated into cancer-associated fibroblasts (CAFs) demonstrated that stromal components significantly alter tumor stem-cell composition and reduce sensitivity to TMZ and radiation (Oliver et al., 2023). Bioprinted 3D systems showed that MSC/CAF interactions promote transcriptomic and epigenetic alterations linked to oxidative phosphorylation and tumor cell survival (Oliver et al., 2024). Supporting the tumor stroma’s pro-resistance role, Zuo et al. (2025) identified CAFs as major sources of Chemokine ligand 2 (CCL2), which promotes activation of the Extracellular Signal-Regulated Kinase 1/2 (ERK1/2) pathway in GBM cells and increases TMZ resistance. Similarly, Mendieta et al. (2025) showed that co-cultures with astrocytes exhibit greater TMZ resistance, associated with biomechanical alterations.

Studies investigating the role of TLS polymerase κ (POLκ) in *MGMT*-deficient GBM models provided additional evidence that 3D architecture affects TMZ response. Using 2D cultures of U-251MG cells, Latancia et al. (2024) demonstrated that TLS Polκ deficiency (by CRISPR/Cas9) increased TMZ sensitivity, enhanced DNA damage accumulation, and altered cell cycle progression following TMZ exposure. Extending these findings to 3D spheroid models, Ribeiro et al. (2024) showed that the same U-251MG TLS Polκ knockout reduced spheroid growth and viability while increasing TMZ-induced cytotoxicity and DNA damage in 3D. However, higher TMZ concentrations were required to achieve comparable responses. Importantly, the 3D spheroid models exhibited resistance compared with monolayer cultures, reinforcing the contribution of tumor architecture and microenvironmental complexity to DNA damage response and DNA damage tolerance pathways in TMZ chemoresistance.

Beyond cellular and structural interactions within the TME, researchers have investigated several molecular pathways associated with therapeutic resistance in 3D GBM models. Pharmacological inhibition of NF-κB potentiated TMZ effects by reducing proliferation, migration, and cytoskeletal reorganization in 3D cultures (Avci et al., 2020). MiR-329 and miR-449b increased TMZ sensitivity by inhibiting Focal Adhesion Kinase (Src/FAK), NF-κB, and Cyclin D1 signaling, thereby reducing proliferation, migration, and spheroid formation (Mendieta et al., 2025). Furthermore, Sha et al. (2025) combined TMZ with PARP inhibitor olaparib and the Nicotinamide Phosphoribosyltransferase (NAMPT) inhibitor FK866, demonstrating tumor suppression in TMZ-resistant cells and organoids, accompanied by NAD + depletion.

Additional mechanisms associated with tumor persistence and metabolic adaptation have also been identified in 3D GBM models, such as (i) the non-coding RNA HSD52 (Sun et al., 2025); (ii) the activation of the Protein Phosphatase 1 Regulatory Subunit 15B/Eukaryotic Translation Initiation Factor 2 Alpha (PPP1R15B/EIF2α) axis (Chuang et al., 2025); and (iii) Glutathione Peroxidase 8 (GPX8) reducing mitochondrial oxidative stress (Yu et al., 2026).

The development of microfluidic technologies and 3D bioprinting systems has considerably expanded the ability to reproduce complex aspects of the TME, including intratumoral heterogeneity, vascularization, spatial gradients of therapeutic resistance, and invasive dynamics. Hydrogel-based microfluidic systems reproduced dose-dependent responses to TMZ and carmustine under biomimetic conditions (Clancy et al., 2022). Pun et al. (2024), using vascularized microphysiological systems, showed that regions adjacent to vascular interfaces exhibit greater TMZ resistance and increased expression of tumor stem cell markers, such as Cluster of differentiation 133 (CD133), reinforcing the contribution of perivascular niches to TMZ resistance. Furthermore, multicompartment bioprinting platforms and Tumoroid-on-a-Plate systems demonstrated that ECM composition modulates tumor invasiveness and TMZ response (Lee et al., 2024; Seyfoori et al., 2025).

This review also showed that novel scalable platforms for pharmacological screening have expanded the translational potential of 3D GBM models. Paper-based systems that can generate, cryopreserve, and reuse tumor spheroids at scale showed high reproducibility in evaluating TMZ cytotoxicity, facilitating parallel drug screening and the development of 3D tumor biobanks (Glia et al., 2025). Furthermore, gelatin/polyethylene glycol microgels containing GBM cells reproduced TMZ responses comparable to those observed in macroscopic hydrogels, thereby enabling integration with automated, high-throughput drug screening platforms and increasing the translational applicability of 3D models (Payan et al., 2026).

### 5.6 Ideal 3D GBM models and TMZ sensitivity

Although three-dimensional models are contemporary, they face notable limitations in clinical research. Avera and Kim (2025) examined stem cell-derived GBM organoids from a bioengineering standpoint, emphasizing the need to overcome scale constraints and develop sophisticated perfusion and bioreactor systems that can reliably support millimeter-sized constructs. Besides these technological hurdles, assessing TMZ sensitivity in 3D models requires accounting for both the intrinsic molecular features of tumor cells—such as MGMT and MMR status—and the effects of the culture system itself. Features like cellular organization, microenvironment interactions, and drug penetration in 3D can influence response, making it hard to separate these factors when comparing cell lines or studies. The most accurate approach is to compare the same cellular model in both 2D and 3D conditions. While 3D culture can induce more treatment-resistant phenotypes, this is not always the case and depends on the specific cellular background and experimental setup. They highlighted the need to overcome scale limitations and develop advanced perfusion and bioreactor systems that can reliably sustain millimeter-scale constructs.

To evaluate TMZ response, utilize a 3D GBM model that balances biological relevance with reproducibility and aligns with the experimental goal. Important factors include the cellular origin and molecular background, especially *MGMT* and MMR status; spheroid or organoid size; culture duration; and relevant microenvironmental components. Clearly report TMZ concentration and exposure duration, and when possible, include clinically relevant exposure ranges, as supraphysiological levels may hinder translational relevance. Use consistent treatment schedules and response evaluations, and supplement viability measures with other parameters such as 3D growth, cell death, proliferation, or long-term regrowth when suitable. Quantitative response data, such as concentration–response curves and IC50 values, can aid cross-study comparisons. Rather than relying on a single “ideal” 3D model, adopting standardized, purpose-fit approaches can improve reproducibility and the translational potential of TMZ sensitivity research.

## 6 Current challenges and future directions

GBM remains a major clinical and scientific challenge, as critical gaps persist in understanding the factors that trigger its development and the mechanisms that ultimately determine therapeutic response and resistance. Thus, establishing reliable, replicable study models and protocols is essential to enable further investigation.

Despite significant advances in 3D *in vitro* models, several challenges still limit their application and translational potential. A major barrier is the lack of standardization across experimental protocols, including differences in cell sources, scaffold composition, and culture conditions, which directly affect reproducibility and comparability between studies.

In this context, the continuous refinement of experimental models is indispensable. Advanced 3D systems, particularly PDOs and integrated platforms such as organs-on-a-chip or bioprinted constructs, enable individual assessments and the investigation of treatment options, offering the most promising opportunities. However, GBM heterogeneity is a major challenge for developing reproducible models and achieving therapeutic responses. At the intratumoral level, individual tumors comprise distinct cellular populations and transcriptional states with different properties and treatment sensitivities, and this heterogeneity likely contributes to the failure of conventional and targeted therapies to achieve long-term remissions (Patel et al., 2014). At the intertumoral level, tumors from different patients may vary in genetic alterations, cellular composition, and molecular profiles. PDOs can recapitulate important aspects of both intra- and intertumoral heterogeneity; nevertheless, this variability may directly impact model establishment and experimental outcomes (Jacob et al., 2020). Incorporating additional components, such as immune cells and vasculature, is a critical step toward more physiologically relevant models. As these models become more representative, they may help identify therapeutic vulnerabilities, patient-specific response patterns, and clinically relevant treatment thresholds. In the future, the growing availability of patient-derived samples, combined with advances in 3D modeling, may support the validation of more effective therapeutic interventions for GBM.

## 7 Conclusion

In summary, this review highlights the growing importance of 3D *in vitro* models as tools for investigating mechanisms of TMZ chemoresistance and for developing new therapeutic strategies in GBM. The findings summarized here show that TMZ response depends heavily on cell type (including MGMT promoter methylation status) and on the structural complexity of the 3D system (scaffold-free and/or scaffold-based), with higher concentrations required for more elaborate models, such as microfluidic devices or organs-on-chip. These results more accurately reflect clinically relevant resistance patterns because they employ cells and models that more closely mimic the vascular system (endothelial cells) and the ECM (fibroblasts and synthetic and natural matrices). Strategies such as knocking out resistance-related genes, including translesion polymerase *Polκ*, Glial Cell Line-Derived Neurotrophic Factor *(GDNF),* GDNF Family Receptor Alpha 1 *(GFRA1),* Mitogen-Activated Protein Kinase 1 *(MAP3K1)*, and *HSD52*, have also been explored and appear promising for overcoming resistance.

These reviewed 3D culture systems represent a powerful and evolving platform for GBM research, especially patient-derived models like PDOs and PDGCs, which were highly represented in this review; IPTOs remain underexplored. The patient-derived models obtained from surgically resected GBM tissues have promising applications for functional assays, drug screening, and personalized treatment (Hou et al., 2022). Continued technological innovation in these organoids and methodological refinement may improve reproducibility, scalability, and predictive value, expanding their applicability to developing new GBM drugs and individualized therapies based on patients’ tumor profiles.
