# Supplementary material for: Three-dimensional in vitro models for assessing temozolomide sensitivity in glioblastoma
Source: Front Neurosci. 2026 Oct 21;20:1901494. doi: 10.3389/fnins.2026.1901494 (PMC13732461; doi:10.3389/fnins.2026.1901494)
Supplement: Supplementary file 1 [file Table_1.docx]

**Supplementary Table 1:**

| **Cell line** | **3D model** | **TMZ concentrations** | **Time of treatment** | **Assays** | **Main Findings** |  |
| --- | --- | --- | --- | --- | --- | --- |
| **Scaffold-free** | | | | | | |
| U-251MG and A-172 | Scaffold-free (ULA) | 25 - 200 μM | 72 h | - Cell viability (acid phosphatase assay)  - Spheroid migration | - TMZ did not affect the viability or migration of the cells | [(Rodrigues-Junior et al., 2022)](https://www.zotero.org/google-docs/?l6d39G) |
| iPSC  (organoid) | Scaffold-free (ULA) | 700 μmol/well | 120 h | - Immunofluorescence (CASP3/beta III tubulin) | - TMZ ↑ caspase-3 and anti-beta III tubulin in *c-met-*mutated iPSC-A | [(Hwang et al., 2020)](https://www.zotero.org/google-docs/?d8whiI) |
| PDOs | Scaffold-free (ULA) | 500 µM | 72 h | - Cell cycle (FC)  -Protein (Immunoblotting)  and gene expression (RT-qPCR)  - DNA methylation (MS-PCR) | - ↓ number of cells in G2/M phase after TMZ treatment  - TMZ did not change the expression of genes involved in the G2/M phase  - Independent of the *MGMT* status, TMZ-induced G2/M arrest and activation of the caspase cascade | [(Ciechomska et al., 2020)](https://www.zotero.org/google-docs/?sfMNuf) |
| LN-229 and co-culture with astrocytes | Scaffold-free (ULA) | 600 µM | 7 d | - Cell viability (Trypan blue)  - Spheroid area  - Apoptosis (Tunel assay)  - Gene (RT-qPCR) and protein (Western Blot) expression | - Cell viability in LN-229 ↓ compared with the co-culture and astrocyte monoculture treated with TMZ  - TMZ ↓ spheroid size in co-culture and LN-229  - TMZ ↑ TUNEL (+) cells in co-culture and LN-229 spheroids  - TMZ ↓ GFAP, vimentin, Notch-1, and survivin in co-culture and LN-229 spheroids | [(Pustchi et al., 2020)](https://www.zotero.org/google-docs/?myXDDA) |
| U-87MG and LN-229 | Scaffold-free (ULA) | 100 μM | 7 d | - Cell viability (calcein-AM and PI) | - Smaller spheroids (~150-300 μm) were responsive to TMZ, showing a significant ↓ in size | [(Singh et al., 2020)](https://www.zotero.org/google-docs/?vcp7y1) |
| U-87MG, LN-229 and PDOs | Scaffold-free (ULA) | 50-200 µM | 7 d | - Cell viability (PI and Ho)  - Autophagy (AO)  - Protein expression (Immunoblotting) | - Cell death was induced only at TMZ 200 µM  - TMZ 50 µM ↑ AO staining (autophagy)  - ↑ conversion of LC3-II along with an ↑ in LC3-I levels, loss of p62, ↑ ATG3, ATG6 and ATG7 | [(Abbas et al., 2020)](https://www.zotero.org/google-docs/?uewlOn) |
| NCH644, GBM1, BTSC233, JHH-520, SF188 | Scaffold-free (Tumorspheres) | 5 - 100 µM | 6 d | - Cell viability (MTT assay) | TMZ IC50: NCH644: 110 µM, GBM1: 5 µM, BTSC233: 10 µM, JHH-520: 10 µM, SF188: 40 µM | [(Vargas-Toscano et al., 2020)](https://www.zotero.org/google-docs/?yyXsKP) |
| U-87MG and PDOs | Scaffold-free (Tumorspheres) | 250 μM | 72 h | - ATP levels (CellTiter-Glo)  - Cell viability (WST)  - Spheroid area  - Invasion assay  - Protein (Immunocytochemistry, Western blot) and gene expression (microarray) | - TMZ ↓ proliferation of U-87mg and PDOs  - ↓ ATP levels only in U-87MG  - ↓ formation of spheroids  - ↓ stemness markers (CD133, nestin, PDPN, Oct3/4)  - presented an anti-invasive effect  - ↓ transition and invasion markers (N-cadherin, Snail)  - ↓ stemness- and invasiveness-related genes | [(Park et al., 2021)](https://www.zotero.org/google-docs/?RfAr7S) |
| PDOs | Scaffold-free (ULA) | 10 µM | 7 d | - Cell viability (CellTiter-Glo)  - Spheroid area  - Senescence/apoptosis (FC)  - Invasion  - Immunofluorescence (MitoTracker, MitoSOX, LysoTracker) | - TMZ had no impact on viability and morphology  - TMZ accelerated MMP signals  - TMZ did not boost hypoxia  - TMZ was not able to prevent invasiveness | [(Riess et al., 2021)](https://www.zotero.org/google-docs/?goEmid) |
| U-251MG, U-87MG, T98G | Scaffold-free (ULA) | 40 and 72 μM | 72 h | - Spheroid area  - Protein expression (Immunohistochemistry)  - CellROX® Green reagent  - ELISA (8-OHdG level)  - Immunofluorescence (8-oxo-dG) | - TMZ (40 μM): not cytotoxic and did not inhibit spheroid area  - U-251MG (TMZ 40 μM): ↓ Ki-67+ cells  - U-87MG (TMZ 72 μM): ↑ intracellular ROS  - U-251MG (TMZ 40 μM): ↓ GSH levels  - TMZ ↓ GPX4 expression | [(Shaw et al., 2021)](https://www.zotero.org/google-docs/?mPNkFM) |
| PDOs | Scaffold-free (ULA) | 30 µM | 48 h | - Cell cycle (FC)  - Cell death (PI)  - Cell proliferation (Clonogenic survival) | - TMZ did not exert any effect on the cell cycle  - TMZ ↑ sub-G1 fraction  - TMZ did not ↓ plating efficiency | [(Zirjacks et al., 2021)](https://www.zotero.org/google-docs/?SaqK8O) |
| U-251MG | Scaffold-free (Hanging Drop) | 100 µM | 72 h | - Spheroid area  - Cell migration | - There were no changes in the size of the spheroids treated with TMZ  - TMZ did not ↓ the migration area | [(Scheffel et al., 2022)](https://www.zotero.org/google-docs/?tvBYZJ) |
| U-87MG and HUVEC | Scaffold-free (ULA) | 100 μM | 48 h | - Spheroid area  - Gene (RT-qPCR) and protein expression (Immunofluorescence)  - Cytotoxicity (Live/Dead Cell Assay) | - Co-culture spheroids became more compact over a period of 7 days  - TMZ ↓ spheroid size and viability  - TMZ ↑ NANOG, and OCT4 (gene and protein) | [(Nakod et al., 2022)](https://www.zotero.org/google-docs/?TPMktb) |
| PDOs | Scaffold-free (ULA) | 50 - 400 µM | 72 h | - Cell viability (Presto Blue)  - Cell cycle (FC)  - RT-qPCR | - TMZ cytotoxicity ↓ in spheroids maintained at 5% O2  - At 5% and 20% of O2, there is no difference in the expression of cell cycle genes and cell cycle distribution | [(Gozdz et al., 2022)](https://www.zotero.org/google-docs/?xMCyoq) |
| U-87MG | Scaffold-free (ULA) | 100 μM | 8 d | - Spheroid area  - Cell viability (LIVE/DEAD assay) | - ↓ spheroid size  - ↓ viability | [(Yadav et al., 2022)](https://www.zotero.org/google-docs/?mQPvcO) |
| PDOs | Scaffold-free (ULA) | 150 µM | 7 d | - Spheroid area | - No gliomaspheres with a diameter bigger than 50 μm were observed | [(Rodrigues-Junior et al., 2022)](https://www.zotero.org/google-docs/?7MqZ0R) |
| T98G and A-172 | Scaffold-free (ULA) | - 1000 µM: T98G  - 900 µM: A-172 | 96 h | - Spheroid area  - Cell viability (Calcein-AM/PI staining)  - Gene expression (RT-qPCR) | - TMZ ↓ area in both cell lines  - TMZ ↓ viability in both cell lines  - ↓ marker genes *CD133* (*PROM1*) and *OCT4* in both cells | [(Ercelik et al., 2023b)](https://www.zotero.org/google-docs/?jhCUCj) |
| U-87MG | Scaffold-free (ULA) | 7.8 - 750 µM | 72 h | - Cell viability (acid phosphatase assay)  - Spheroid area | - TMZ (750 µM) ↓ 5% of spheroids' viability  - ↑ TMZ concentrations ↓ growth | [(Erthal et al., 2023)](https://www.zotero.org/google-docs/?KSndTm) |
| Primary human astrocytes transfected with HCMV | Scaffold-free (ULA) | 50 µM | 10 d | - Spheroid area | TMZ ↓ spheroids size (23%) in CEGBCs-BL cultures but not in CEGBCs-DB, which displayed more mesenchymal traits | [(El Baba et al., 2023)](https://www.zotero.org/google-docs/?Zm1vb0) |
| U-3054 | Scaffold-free (ULA) | 48 µM | 3 and 5 d | - Invasion assay | - TMZ did not affect the invasion under hypoxia and normoxia | [(Sousa et al., 2023)](https://www.zotero.org/google-docs/?O9fNvi) |
| U-87MG | Scaffold-free (ULA) | 250 μM | 48 h | - Cell viability (BCECF and Sulforhodamine B probes)  - Spheroid area | - TMZ presented higher toxicity at pH = 7.4 | [(Tafech et al., 2023)](https://www.zotero.org/google-docs/?ppxja9) |
| U-118MG, U-138MG, U-87MG, U-87MG-RFP-Luc | Scaffold-free (ULA) | 0.01-1000 µM | 70 h | - Spheroid area  - Cytotoxicity (Sytox™ green Dead Cell Stain) | - U118MG showed the highest sensitivity to TMZ, folowed by U-87MG and U-118MG for both growth and cytotoxicity | [(Davy et al., 2023)](https://www.zotero.org/google-docs/?KOjFF1) |
| PDOs  (Organoid) | Scaffold-free (ULA) | 800 µM | 5 d | - Organoid area | - The architecture of organoids exhibited a relaxed and even disintegrated after TMZ  - TMZ decreased the organoid area | [(Qu et al., 2023)](https://www.zotero.org/google-docs/?a6BWiX) |
| T98G | Scaffold-free (ULA) | 1000 μM | 120 h | - Spheroid area  - Cell viability (Calcein-AM/PI) | - TMZ ↓ T98G sphere size  - TMZ ↓ cell viability and ↑ necrotic cells | [(Ercelik et al., 2023a)](https://www.zotero.org/google-docs/?7aRVWz) |
| PDOs and U-87MG | Scaffold-free (Hanging Drop) | 200–1000 μM | 17 d | - Spheroid area | - U-87MG spheroid growth is very slow, even in the absence  - TMZ in all concentrations ↓ area | [(Oraiopoulou et al., 2024)](https://www.zotero.org/google-docs/?Ep8S5H) |
| U-87MG | Scaffold-free (ULA) | TMZ nanoparticles (5-500 μg/mL) | 24h | - Cell viability (CellTiter-Glo) | - TMZ nanoparticles IC50 of 213 μg/mL  - Non-encapsulated TMZ IC50 of 560 μg/mL | [(Cai et al., 2024)](https://www.zotero.org/google-docs/?qGiGiI) |
| U-251MG WT and U-251MG TLS Polκ KO | Scaffold-free (ULA) | 25 - 200 µM | 24, 48, 72, 96, and 120 h | - Cell viability (resazurin and CellTiter-Glo)  - Spheroid area  - Proliferation (clonogenic assay)  - Genotoxicity (53BP1trunc-apple plasmid)  - Cell cycle (Fast-FUCCI plasmid)  - Cell death (Caspase 3/7)  - Migration and invasion | - ↑ cytotoxicity in KO cells  - TMZ ↓ spheroid area in KO cells  - TMZ ↓ proliferation and promotes cell cycle arrest (G2/M phase) in KO cells  - KO spheroids ↑ cell death and genotoxicity after TMZ exposure  - TMZ ↓ invadopodia more effectively in Polκ KO spheroids | [(Ribeiro et al., 2024b)](https://www.zotero.org/google-docs/?JIjqmJ) |
| U-87MG | Scaffold-free (ULA) | 0.15 mg (liposomes) | 72 h | - Cell viability (CellTiter-Glo)  - Spheroid area | - TMZ liposome/gel group ↓ cell viability and diameter  - TMZ liposome/gel, TMZ gel, and TMZ liposome ↓ diameter of the 3D spheroids | [(Yue et al., 2024)](https://www.zotero.org/google-docs/?aPlfv3) |
| PDOs (GDNF KO and GFRA1 KO) | Scaffold-free (ULA) | 50 μM and 100 μM | 48 h | - Gene expression (RNA-Seq)  - Cell viability (MTT)  - CRISPR/Cas9 | - ↑ of *GDNF* and *GFRA1* after TMZ (50 μM) GDNF-KO cells ↓ growth compared to *GDNF*-expressing cells  - *GDNF* KO↑ sensitivity to chemotherapy  - ↑ Sensitivity was reversed by the addition of GDNF | [(Avenel et al., 2024)](https://www.zotero.org/google-docs/?bXVP5p) |
| PDOs (organoid) | Scaffold-free (Tumorspheres) | 50 μM | Every 48 h for 7d | - Cell viability (Cell-Titer Glo)  - Invasion  - Apoptosis (annexin-V-FITC)  - Gene expression (RT-qPCR) | - TMZ does not affect organoid viability or invasion, but affects apoptosis  - TMZ ↑ *MDM2*, *ATM*, *CD44*, and *ATR* | [(Majc et al., 2024)](https://www.zotero.org/google-docs/?QcqmQv) |
| LN-18, LN-229 and PDOs | Scaffold-free (ULA) | 100 µM | 7 d | - Cell viability (calcein-AM, Helix NPTM Blue, and PI) | - Larger spheroids potentially showing no difference in size following treatment with TMZ  - TMZ ↓ cell viability at 72h | [(Phour and Vassella, 2024)](https://www.zotero.org/google-docs/?84IasE) |
| PDOs (organoid) | Scaffold-free (ULA) | 200 μM | 21 d | - Organoid area  - Cell viability (Live‐Dead) | - MAP3K1 KO combined with TMZ ↓ the growth and ↑ cell death | [(Wu et al., 2024)](https://www.zotero.org/google-docs/?VZKR9x) |
| U-87MG | Scaffold-free (ULA) | 100 μM | 5 d | - Spheroid area | - TMZ-sensitive spheroids ↓ area and stop growing in fifth day, maintaining their size for the rest of the experiment  - TMZ-resistant ↓ area until day 21growth | [(Pérez-Aliacar et al., 2024)](https://www.zotero.org/google-docs/?BzIKXs) |
| PDOs co-culture with microglia | Scaffold-free (Tumorspheres) | 60-120  μM | 24-72 h | - Cell death (PI assay)  - Cell viability (WST‐1)  - Protein (Immunohistochemistry) and gene expression (RNA-Seq) | - Microglia ↓ GBM cell death  - Microglia ↑ STAT1 signaling pathway  - Microglia ↑ expression of ISGs and *CDKN1A*  - Microglia ↑ pro-apoptotic factors | [(Sørensen et al., 2024)](https://www.zotero.org/google-docs/?3nsx7d) |
| A-172, H4, U-118MG, U-87MG, and U-373MG | Scaffold-free (ULA) | 251 μM | 72 h | - Cell viability (resazurin)  - Spheroid area  - MCTs invasion assay  - Gene expression (RT-qPCR) | -IC50 values: 197 to 639 μM (U-373MG < U-87MG < A-172) at 48 h and 144–554 μM (U-373MG < U-87MG < A-172) at 72 h  - TMZ (251 μM) ↓ spheroid size  - TMZ did not ↓ invasion but may contribute to it.  - TMZ ↓ *BCL2* mRNA | [(Basso et al., 2024)](https://www.zotero.org/google-docs/?YPX7jx) |
| U-87MG | Scaffold-free (magnetic levitation) | 10 - 1500 µM | 120 h | - Cell viability (CCK‐8)  - Spheroid area  - ATP‐based luciferase assay | - IC50 is 669.2 µM  - TMZ ↓ spheroid area  - TMZ did not change ATP levels | [(Datta et al., 2024)](https://www.zotero.org/google-docs/?ZwoiZP) |
| G55T2, U-87MG, and  U-343MG | Scaffold-free (ULA) | 200 µM | 5 d | - Cell viability (ATP levels and CellTiter-Glo)  - Spheroid area | - TMZ ↓ cell viability in all 3 cell lines  - TMZ ↓ spheroid area in the 3 cell lines | [(Bach et al., 2024b)](https://www.zotero.org/google-docs/?8UYizI) |
| U-251MG and T98G | Scaffold-free (ULA) | 2000 μM | 6 d | - Spheroid area | - TMZ ↓ spheroid area in T98 but not in U-251MG spheroids | [(Oliveira et al., 2025)](https://www.zotero.org/google-docs/?uUm9sR) |
| iPSCs  (organoid) | Scaffold-free (ULA) | 50 μM | 10 d | - Cell viability (Bioluminescence)  - Malignant cell state (NPC, MES)  - Gene expression (scRNA-seq) | - One organoid was responsive to TMZ and two were not  - TMZ ↓ NPC-like tumor cells in all three samples  - TMZ ↑ C-like and MES-like tumor cells  - Resistent organoids had ↑ SPP1, FTL, CD74, CTSB, and CLU | [(Peng et al., 2025b)](https://www.zotero.org/google-docs/?RDPe5n) |
| U-87MG and U-118MG | Scaffold-free (Hanging Drop) | 10 µM | 72 h | - Cell viability (PrestoBlue assay)  - Cell death (Annexin V-FITC/PI)  - Spheroid area | - ↓ viability in both cell lines  - ↑ apoptosis at 24 h  - U-118MG: ↓ necrotic cells  -U-87MG: ↑ necrotic cells at 48 h  - ↓ spheroid size | [(Bellavita et al., 2025)](https://www.zotero.org/google-docs/?ClYOeC) |
| PDOs | Scaffold-free (Tumorsphere) | 5 –175 µM | 6 d | - Tumor-spheres forming ability  - Cell viability (MTT assay) | - Tumorspheres were formed in 6 d  - IC20 TMZ was: BT453: 100 µM; BT314: 175 µM; BT302: 5 µM; BT138: 25 µM | [(Díaz Méndez et al., 2025)](https://www.zotero.org/google-docs/?GvoDoV) |
| LN-229, PDM-140, U-87 | Scaffold-free (ULA) | 1-1000 µM | 7 d | - Spheroid area  - Cell viability (PrestoBlue) | - TMZ ↓ viability LN-229 (100µM), in 1000 µM viability was 8.52%  - ↓ viability PDM-140 (50µM), in 100 µM (30.4%)  -↓ viability U-87 (250µM), in 1000 µM (26%)  - TMZ ↓ spheroid area in LN-229, PDM-140 and U-87 | [(Maity et al., 2025)](https://www.zotero.org/google-docs/?AzJpQP) |
| U-251MG | Scaffold-free (ULA) | 0.976 µM | 48 e 144 h | - Cell viability (Alamar Blue™, PI) | - An IC50 of 133.0 µM was found for TMZ after 144 h and 300.1 µM after 48h | [(Wanigasekara et al., 2025)](https://www.zotero.org/google-docs/?yujsBo) |
| U-87MG | Scaffold-free (hanging drop) | 625 μM | 48 h | - Spheroid area  - PI | - TMZ has not shown any significant reduction in the size of the spheroids | [(Dey et al., 2025)](https://www.zotero.org/google-docs/?RGjNuj) |
| PDOs | Scaffold-free (Tumorsphere) | 250 - 1000 μM | 72 h | - Cell viability (WST-8/CCK8)  - ATP levels (CellTiter-Glo)  - Protein expression (Western blot) | - TMZ ↓ viability (concentration-dependent) and ATP levels from fresh and cryopreserved tissues  - TMZ (500 μM) ↑ *BAX* and ↓ *BCL2* and *PARP*  - TMZ ↑ apoptosis  - Cryopreserved tissues are suitable for TMZ efficacy | [(Na et al., 2025a)](https://www.zotero.org/google-docs/?7wkWqv) |
| PDOs  (organoid) | Scaffold-free (ULA) | 1000 μM | 4 d | - Spheroid morphology  - Cell death (SYTOX Green staining) | - TMZ induced morphological changes  - ↑ in SYTOX Green‐positive cells indicating a cytotoxic effect | [(Ye et al., 2025)](https://www.zotero.org/google-docs/?nDMl6g) |
| U-87MG | Scaffold-free (ULA) | 100 μM | 5 d | - Spheroid area | - TMZ ↓ spheroid growth | [(Chantaravisoot et al., 2025)](https://www.zotero.org/google-docs/?QPlwch) |
| U-87MG | Scaffold-free (Hanging Drop) | 25 - 500 μM | 48 h | - Cell viability (APH assay)  - Spheroid area | - ↓ viability (time- and dose-dependent)  - IC50 values were 1420 μM at 48 h and 956 μM at 72 h  - TMZ (500 μM) ↓ growth and viability by the third treatment | [(Aksüt et al., 2025)](https://www.zotero.org/google-docs/?WCxIc2) |
| PDOs  (organoid) | Scaffold-free (Agitation-based culture) | 100 µM | 144 and 288 h | - Total cell count  - Cell viability (LDH assay)  - Cell death (PI and Ho, flow cytometry)  - Organoid area | - TMZ ↓ cell number  - TMZ ↑ LDH release of damaged cells  - ↑ cell death in MGMT-methylated organoids but not in MGMT-unmethylated  - TMZ slight ↓ diameter over 14 days | [(Potthoff et al., 2025)](https://www.zotero.org/google-docs/?pwJNBA) |
| U-87MG | Scaffold-free (ULA) | 100 μM | 24 h | - Spheroid area  - Cell death (PI)  - Immunofluorescence (Ki‐67) | - TMZ ↓ cell death  - ↓ spheroid size  - TMZ did not change Ki67 | [(Mannino et al., 2025)](https://www.zotero.org/google-docs/?3tL8qi) |
| U-87MG | Scaffold-free (ULA) | 0.001 - 1000 μM | 6 d | - Spheroid area | - IC50 of TMZ was 260 μM | [(Colpan et al., 2025)](https://www.zotero.org/google-docs/?CX0nBj) |
| U-87MG | Scaffold-free (Tumorsphere) | 211 µM | 48 h and 5d | - Spheroid area  - Gene expression (RT-qPCR)  - NO levels | - After 2–5 d, TMZ disaggregated spheroids into smaller clusters  - ↓ *CTNNB1, SNAIL2, ITGA5,* and *TRPM7* | [(Tehrani et al., 2025)](https://www.zotero.org/google-docs/?uJYhRP) |
| PDOs | Scaffold-free (ULA) | 50 and 100 µM | 72 h | - Cell viability (CCK-8)  - Spheroid migration | - TMZ ↓ migration and cell viability in a dose-dependent manner | [(Khotchawan et al., 2025)](https://www.zotero.org/google-docs/?aio2eL) |
| LN-229, U-87MG, U-251MG and PDOs | Scaffold-free (Hanging Drop) | 500-1000µM | 5 d | - Spheroid area  - Gene expression (RT-qPCR)  - NO levels | - TMZ ↓ area in all cell lines and PDOs  - TMZ ↑ Nestin (pluripotency marker)  - TMZ had a tendency to ↑ NO levels  - The SFE ↓ after TMZ in all cell lines but not in PDOs | [(Hincapié-Arias et al., 2025)](https://www.zotero.org/google-docs/?uDpxnE) |
| PDOs | Scaffold-free (ULA) | 200 μM | 14 d | - Organoid area  - Cell viability (CCK8)  - Apoptosis (FC)  - Protein expression (Immunofluorescence) | - TMZ ↓ organoid size and proliferation  - TMZ ↓ ki67  - ↑ COL6A1 in organoids resistents to TMZ | [(Gao et al., 2026)](https://www.zotero.org/google-docs/?kc3pzR) |
| U-87MG, A-172 | Scaffold-free (ULA) | 100 μM | 7 d | - Spheroid area  - Cell death (PI) | - TMZ ↓ spheroid area on day 7  - TMZ preserved spheroid architecture | [(Scuderi et al., 2026)](https://www.zotero.org/google-docs/?ZYtq7v) |
| PDOs, DK-MG and HMC3 | Scaffold-free (ULA) | 200 and 400 µM | 24 or 48 h | - Spheroid area  - Cell viability (Flow cytometry) | - DKMG homotypic ↓ size after 24 h TMZ (200 µM), recovered after 48 h  - DKMG heterotypic maintained a larger size  - GB22-13 heterotypic ↓ size after 400 µM TMZ for 24 h, recovered after 48 h  - The percentage of GB22-13 viable cells ↓ after TMZ | [(García-Sáez et al., 2026)](https://www.zotero.org/google-docs/?OPA6MM) |
| HEK-293-T and BT-379 (GSCs) cells (GLICO) | Scaffold-free (ULA) | 100 μM TMZ | 7 d | - Cell viability (fluorescence) | - TMZ ↓ the tumor growth of shMEOX2 GLICO model | [(Russo et al., 2026b)](https://www.zotero.org/google-docs/?hHwfzY) |
|  |  |  |  |  |  |  |
| **Scaffold-based** | | | | | | |
| PDOs and HK301 | Scaffold-based (Hydrogel) | 500 μM | 12 d (2 cyles of 3 d treatment and 3 d of rest) | - Cell viability (luciferase)  - Protein expression (Western blott)  - Cell proliferation (Flow cytometry) | - TMZ ↓ cell viability in both spheroids  - Cytostatic effect in both spheroids  - ↑ PARP clivage in both spheroids  - TMZ ↓ viability in CD44KO or integrin αV spheroids  - ↑ PUMA, BAX, and BAK in HK301 and ↓ in PDOs  - In HK301 and GBM6, pro-apoptotic factors expression was consistent with CD44 and integrin αV KO | [(Xiao et al., 2020)](https://www.zotero.org/google-docs/?vggXwK) |
| U-87MG and U-251MG | Scaffold-based (Microfluidic) | 100 μm | 72 h | - Cell viability (calcein AM)  - Live/dead assay (DAPI, LC3-GFP, P62) | - TMZ ↓ viability  - TMZ ↑ number of cells with cleaved *PARP*  - ↑ apoptosis and ↓ autophagy flux | [(Shojaei et al., 2020)](https://www.zotero.org/google-docs/?NfsKY1) |
| U-251MG and U-87MG | Scaffold-based (Microfluidic + Hydrogel) | 100 - 500 µM | 72 h | - Cell viability (Live/Dead)  - Protein expression (Immunofluorescence)  - Invasion | - ↓ viability according TMZ concentrations in both cells  - ↑ PARP and Caspase 3 cleavage in U-251 cells  - U-251 cells were more sensitive than U-87 cells  - ↑ LC3β and SQSTM1/p62 (autophagy markers)  - ↓ U-251 and U-87 invasion | [(Samiei et al., 2020)](https://www.zotero.org/google-docs/?8fKP8v) |
| LN-229, U-87MG, and PDOs | Scaffold-based (Hydrogel) | 300 µM | 7 d | - Cell viability (Trypan Blue)  - Spheroid area  - Detection of p-p65 by ELISA | - TMZ ↓ viability in LN-229 (66%), U-87MG (59%), and PDOs (82%)  - TMZ ↓ spheroid size in LN-229 (57%), U-87MG (51%), and PDOs (40%)  - TMZ ↓ NF-κB p-p65 activity in LN229 (43%), U-87MG (76%), and PDOs (55%) | [(Avci et al., 2020)](https://www.zotero.org/google-docs/?VhslJr) |
| U-87MG | Scaffold-based (Hydrogel) | 100 μM | 3 d | - Spheroid area  - Cell viability (MTT assay)  - Gene expression (RT-qPCR) | - TMZ showed an anti-proliferative effect (↓area)  - ↓ cell viability  - TMZ ↑ *MGMT* and *ABCB1* | [(Dragoj et al., 2021)](https://www.zotero.org/google-docs/?1XtGIs) |
| PDOs (organoid) | Scaffold-based (Hydrogel) | 250 μM | 6 d | - pH3 (immunohistochemistry) DNA content (CellTracker Blue CMAC Dye)  - Cell viability (CellTiter-Glo)  - Protein expression (Immunofluorescence)  - Senescence | - ↓ pHH3 and mitotic cells  - GBM organoids demonstrated greater overall resistance to TMZ than spheres  - TMZ ↑ Cleaved Caspase-3 (CC-3) and To-Pro3+ apoptotic markers  - TMZ ↑ senescence marker | [(Sundar et al., 2022)](https://www.zotero.org/google-docs/?Kbef04) |
| U-87MG | Scaffold-based (Microfluidic + Hydrogel) | 0.5 - 2mM | 48 h | - Cell viability (PI) | - PEGDA-encapsulated U-87MG: EC50  ~ 0.61 mM  - TMZ in 4-arm PEG-Ac gel: EC50  ~ 1.8 mM  - Cells were less susceptible to TMZ when encapsulated in the degradable, adhesive 4-arm PEG-Ac hydrogel | [(Clancy et al., 2022)](https://www.zotero.org/google-docs/?9zAk9M) |
| PDOs | Scaffold-based (Hydrogel) | 5 - 500 µM | 5 d | - Cell proliferation (EdU) | - TMZ-sensitive cultures were derived from patients with *MGMT* promoter methylation | [(Brown et al., 2023a)](https://www.zotero.org/google-docs/?L0CSIp) |
| PDOs + cancer-activated fibroblasts | Scaffold-based (Hydrogel) | 50 and 100 μM | 96 h | - Cell viability (Cell number)  - MitoT Deep-Red | - TMZ ↓ cells in monoculture compared to the co-cultures: CAFs protected the PDOs from cell death  - TMZ ↓ MitoT Deep-Red fluorescence | [(Oliver et al., 2023)](https://www.zotero.org/google-docs/?Bm6Nng) |
| A-172, BT-169, U-87MG, and PDOs (organoids) | Scaffold-based (Hydrogel) | 10 - 1000 μM | 7 d | - Cell viability (CellTiter-Glo, and Calcein) | - TMZ (1000 μM) killed most of the cells | [(Che et al., 2023)](https://www.zotero.org/google-docs/?Bp747X) |
| U-87MG | Scaffold-based (Hydrogel) | 2 mM | 48 h | - Spheroid area  - Cell viability (DiOC/PI) | - TMZ ↓ area for all hydrogel conditions  - ↓ in soft (1.25), stiff (1.14), and dual-soft (1.37-fold)  - TMZ ↓ viability with no difference between gels | [(Bruns et al., 2023)](https://www.zotero.org/google-docs/?50CPIf) |
| PDOs  (organoid) | Scaffold-based (Hydrogel) | 15 - 300 μM TMZ | - 5 d | - Cell viability (CellTiterGlo)  - Gene expression (RNA Sequencing) | - TMZ resistance at 15 and 30 μM, except for 2 MGMT-methylated organoids  - MGMT-unmethylated organoids were resistant to TMZ and ↓ cell viability at >100 μM  - TMZ ↑ NK-kinase pathway | [(Verduin et al., 2023)](https://www.zotero.org/google-docs/?ZkFBBH) |
| LN-229 | Scaffold-based (Hydrogel) | 300 μM | 7 d | - Cell viability (Trypan-Blue)  - Gene expression (RT-qPCR) | - TMZ ↓ cell viability  - TMZ ↓ spheroid area  - ↑ *BAX* and ↓ *BCL2* expression | [(Mendieta et al., 2023)](https://www.zotero.org/google-docs/?i3zFz7) |
| PDOs  (organoid) | Scaffold-based (Hydrogel) | 300 µM | 31 d | - Immunofluorescence (CC3 and Ki67) | - PDOs ↓ Ki67+ cells and Cas3+ apoptotic cells  - TMZ unaffected co-stained with HOPX and SATB2 | [(Watanabe et al., 2024)](https://www.zotero.org/google-docs/?vSvroE) |
| PDOs (organoid) | Scaffold-based (Hydrogel) | 600 µM | 72 h | - Metabolic activity (FLIM) | - 17 TMZ-non-responsive (65%) and 9 TMZ-responsive (35%) organoids | [(Morelli et al., 2024)](https://www.zotero.org/google-docs/?O0Ettn) |
| PDOs (organoid) | Scaffold-based (Hydrogel) | 500 µM | 120 h | - Immunofluorescence (CC3 and Ki67) | - TMZ ↑ CC3 levels and did not change Ki67 | [(Rampazzo et al., 2024)](https://www.zotero.org/google-docs/?EfjKfd) |
| PDOs + MSC  Organoids | Scaffold-based (Bioprinting) | 50 µM | 96 h | - Protein expression (Immunohistochemistry)  - Cell cycle analysis (FC)  - Gene expression (RNA-seq) | - MSC markers (CD90/THY-1, CD10/CALLA) indicated that MSCs were not in the co-culture scaffolds  - TMZ ↓ cell proliferation more in co-culture  - TMZ ↓ TREM-1 expression  - The effect of TMZ appeared to be less pronounced in monoculture than in co-culture | [(Oliver et al., 2024)](https://www.zotero.org/google-docs/?0S794G) |
| U-87MG + Primary human cells | Scaffold-based (Bioprinting) | 12 mM | 72 h | - Cell viability (DRAQ7, Calcein, and Ho) | - TMZ ↓ viability in the periphery of GlioFlow3D  - The % of dead peripheral cells in GlioFlow3D was similar to that in fibrin hydrogels  - Cells in the perivascular regionhad higher resistance | [(Pun et al., 2024)](https://www.zotero.org/google-docs/?5Sxtan) |
| U-87MG and T98G | Scaffold-based (Bioprinting) | 10 μM to 3 mM | 1, 3, and 7 d | - Cell viability (ReadyProbes™)  - Morphological analysis (CellTracker of green CMFDA dye) | - IC50 values for U-87MG and T98G were 232.43 and 1351.44 μM, respectively  - Dots with a high proportion of T98G cells showed overall cell viability >50%  - Dots with ↑ U-87MG cells had cell viability ↓ 50% | [(Lee et al., 2024)](https://www.zotero.org/google-docs/?GEobzW) |
| U-251MG | Scaffold-based (Microfluidic) | 50 - 500 µM | 72 h | - Invasion  - Cell viability (Presto Blue)  - Cell death (Live/Dead assay)  - Gene expression (RT-qPCR) | - ↓ invasion with ↑ TMZ  - The viability is more affected by TMZ when embedded in reelin compared to collagen  - ↓ 20% and 30% in size after 500 µM TMZ in collagen and collagen‐reelin  - TMZ ↓ *BAX/BCL2* ratio | [(Seyfoori et al., 2025)](https://www.zotero.org/google-docs/?xxzjcY) |
| LN-229 and primary human astrocytes co-culture | Scaffold-based (Hydrogel) | 300 μM | 7 d | - Spheroid area | - LN229 spheroids ↓ stiffness and area  - Co - culture of LN-229 and HA spheroids displayed resistance to the TMZ | [(Mendieta et al., 2025b)](https://www.zotero.org/google-docs/?gPFAp7) |
| - PDOs + DYR0100 (iPSC)  - GFP-positive LN-229R cells with HSD52 KO  (organoid) | Scaffold-based (Hydrogel | 100 μM | 21 d | - Fluorescence microscopy images | - ↓ trend of tumor burden in organoids cocultured with HSD52-KO  - TMZ ↓ tumor burden in the organoids cocultured with HSD52-KO  - HSD52 KO ↓ the malignant growth and TMZ resistance | [(Sun et al., 2025b)](https://www.zotero.org/google-docs/?tZlZ6F) |
| U-87MG | Scaffold-based (Bioprinting) | 0.156 - 2.5 mM | 48 h | - Cell viability (CellTiter-Glo) | - Cytotoxic effect observed at concentrations above 1.25 mM  - IC50 of TMZ: 2.31 mM | [(Cui et al., 2025)](https://www.zotero.org/google-docs/?gnbqx3) |
| PDOs  (organoid) | Scaffold-based (Hydrogel) | 50 µM | 48 h | - Immunofluorescence (NAMPT or Ki67)  - Cell viability (CCK-8)  - Protein expression (Western blot) | - ↓ cell number and volume, as well as the presence of fragmented nuclei  - ↑ Ki67 staining +  - No difference in cell viability | [(Sha et al., 2025)](https://www.zotero.org/google-docs/?XeHfjW) |
| PDOs  (organoid) | Scaffold-based (Hydrogel) | 200 - 3200 µM | 9 d, 20 d-interval, 3 d | - Cell viability (Sulforhodamine B) | - TMZ ↓ cell viability, organoid formation, and cell cycle progression more in sensitive cells than in resistant ones | [(Chuang et al., 2025)](https://www.zotero.org/google-docs/?ETd7uc) |
| PDOs  (organoid) | Scaffold-based (Hydrogel) | 400 μM | 5 d | - Immunofluorescence (CC3)  - Protein expression (Immunohistochemistry) | - GBOs following CAF stimulation exhibited ↓ CC3 after TMZ compared to the GBOs without CAF coculture  - TMZ ↑ the pERK1/2 expression in co-culture | [(Zuo et al., 2025)](https://www.zotero.org/google-docs/?OAlNlW) |
| U-87MG | Scaffold-based (Bioprinting) | 400 and 800 µM | 1, 3, and 7 d | - Cell viability (Live/Dead staining and CellTiter-Glo) | - TMZ 400 μM: ↑ viability in scaffold at 24 h  - By day 7, all groups exhibited recovery, likely due to time-dependent TMZ diffusion  - TMZ 800 μM: ↑ viability at day 1, ↓ at days 3 and 7, suggesting deeper drug penetration and ↑ cell death  - TMZ exposure: Time-dependent ↓ viability; at day 7, ↑ cell death with complete loss of spheroid architecture | [(Sambamoorthy et al., 2026)](https://www.zotero.org/google-docs/?srn7Lb) |
| U-87MG | Scaffold-based (Hydrogel) | 100 μM | 6 d | - Invasion | - TMZ inhibitory effects on spheroid invasion were only marginal | [(Wagner et al., 2026)](https://www.zotero.org/google-docs/?jw1h1n) |
| U-87MG cells transfected with sh-GPX8 (organoid) | Scaffold-based (Hydrogel) | 500 μM | 5 d | - Organoid number  - Organoid area  - Gene expression (qPCR)  and protein (Western blot) expression  - ROS levels | - ↓ sh-GPX8 organoids number treated with TMZ  - *GPX8*KO and TMZ ↓ organoid growth  - TMZ ↓ GPX8 expression in sh-GPX8  - TMZ ↑ ROS levels  - TMZ + GPX8KO ↑ E-cadherin and ↓ mesenchymal markers | [(Yu et al., 2026)](https://www.zotero.org/google-docs/?jtPYeZ) |
| U-87MG | Scaffold-based (Hydrogel) | 10 - 100 μM | 48 h | - Cell viability (Alamar blue) | - TMZ (100 μM) ↓ metabolic activity  - TMZ (20 μM) ↓ metabolic activity on day 9 in encapsulated U-87MG cells in PEG-4MAL | [(Payan et al., 2026)](https://www.zotero.org/google-docs/?uM0048) |
|  |  |  |  |  |  |  |
| **Combination of scaffold-free + scaffold-based models** | | | | | | |
| U-87MG | Scaffold-free (ULA) + Scaffold-based (bioprinting) | 500 μM | 72 h | - Spheroid area | - TMZ ↓ spheroid area | [(Han et al., 2020)](https://www.zotero.org/google-docs/?JckyQQ) |
| U-87MG | Scaffold-free (ULA) and Scaffold-based (hydrogel) | 2 mM | 48 h | - Cell viability (DiOC/PI)  - Spheroid area  - Drug screening | - ↑ TMZ resistance in degradable, adhesive hydrogels  - ↑ diameter in all hydrogels and free-floating spheroids  - Limitations to TMZ penetration alone could not explain the ↑ viability of hydrogel-encapsulated spheroids | [(Hill et al., 2021)](https://www.zotero.org/google-docs/?hCgLvT) |
| T98, U-87MG, and PDOs (organoid) | Scaffold-free (Hanging drop) + Scaffold-based (Hydrogel) | 600 μM | 24, 48, 72 h, 7 d, and 14 d | - Metabolic activity (FLIM)  - Spheroid area | - After 72 h, TMZ ↑ oxidative metabolism typical of a less proliferative state in U-87 spheroids but not in T98  - TMZ ↓ area in U-87 but not in T98 spheroids  - In Porganoids, TMZ ↓ area supporting the FLIM-based metabolic predictions | [(Morelli et al., 2022)](https://www.zotero.org/google-docs/?Ktq82Q) |
| PDOs | Scaffold-based (microfluidic devices) + Scaffold-free (ULA) | 77.2 and 47 μM | 17 d | - Cell viability (LDH assay)  - LactateGlo assay  - Invasion  - Protein expression (Immunofluorescence)  - Kinase activity | - TMZ ↓ LDH activity in ULA e microfluidic  - TMZ affect lactate concentrations only in the microfluidic spheroids  - ↓ Migration ability  - ↑ activity of kinases of ERBB pathway  - ↓ activity of Src family kinases and FAK | [(Fabro et al., 2022)](https://www.zotero.org/google-docs/?fvjGkl) |
| U87-ZsGreen, PDOs, and Human brain microvascular endothelial cells | Scaffold-free (hanging drop) and Scaffold-based (microfluidic devices) | 1–5000 µM | 5 d | - Cell viability (viable ZsGreen-positive cells)  - Protein expression (Mass Spectrometry) | -TMZ IC50 of 1500 µM for U-87 spheroids  - TMZ (1000 µM) ↓ proliferation  - TMZ ↑ cell cycle regulation proteins in U-87 spheroids co-cultured with endothelial cells  - PDOs ↑ tumor progression proteins after co-culture with endothelial cells | [(Lam et al., 2023)](https://www.zotero.org/google-docs/?iQlJEL) |
| LN-229 and U-87MG | Scaffold-based (hydrogel) and Scaffold-free (ULA) | 300 µM | 48 h | - Spheroid area  - Cell viability (Trypan blue)  - Protein expression (Western blot) | - TMZ ↓ 75% area (LN-229)  - U87 remained at 78% initial size after TMZ  - ↓ cell viability to 68% (LN229) and 72% (U87)  - ↓ Cyclin D1 in both spheroids  - LN229: ↓ p-FAK ↑FAK  - U-87: ↓ p-FAK and FAK  - ↑ 17% NF-κB (LN-229) and 97% (U87)  - ↓ BAX/BCL2 ratio in both lineages | [(Mendieta et al., 2025a)](https://www.zotero.org/google-docs/?UvurGD) |
| PDOs (organoid) and PDNs | PDOs- Scaffold-based (Hydrogel)  PDNs- Scaffold-free (ULA) | 100 nM to 100 μM | 90 h | - Cell viability (Incucyte, Cell Titre Blue)  - Organoid Area | - PDN models are more sensitive to TMZ than their PDO counterparts  - TMZ ↓ cell viability in PDN but not in PDOs  - GR50% ranging from 5.58 to 93.56 μM TMZ  - TMZ sensitivity trended toward MGMT promoter methylated models | [(Moore et al., 2026)](https://www.zotero.org/google-docs/?OdehqD) |
| U-87MG | Scaffold-based (Microfluidic) and Scaffold-free (ULA) | 400 e 800 µM | 6, 12, 24, and 48 h | cell viability (WST assay) | - TMZ ↓ cell viability in cryopreserved and non-cryopreserved spheroids | [(Glia et al., 2025)](https://www.zotero.org/google-docs/?EgFtKk) |

Legend: *ABCB1:* ATP-binding cassette subfamily B member 1; APH: acid phosphatase; *ATG3*: autophagy related 3; *ATG6:* autophagy related 6; *ATG7*: autophagy related 7; *ATM*: ataxia-telangiectasia mutated; *ATR:* ataxia telangiectasia and Rad3-related; AVO: acidic vacuolar organelles; *BAX*: BCL2 associated X protein; *BCL2*: B-cell lymphoma 2; CAFs: cancer-associated fibroblasts; CC3: cleaved caspase 3; *CCL5*: chemokine (C-C motif) ligand 5; CD10/*CALLA*: common acute lymphoblastic leukemia antigen; *CD133*/*PROM1*: prominin-1; *CD44:* cluster of differentiation 44; CD74: CD74 molecule; *CD90/THY-1:* thymocyte differentiation antigen 1; *CDKN1A:* cyclin-dependent kinase inhibitor 1A; CLU: clusterin; CMV: cytomegalovirus; *COL6A1:* collagen type VI alpha 1 chain; CRISPR: clustered regularly interspaced short palindromic repeats; *CTNNB1:* catenin beta 1; CTSB: cathepsin B; C-like: classical-like; DiOC/PI: dihexyloxacarbocyanine iodide and propidium iodide; EC50: half maximal effective concentration; ECM: extracellular matrix; EdU: 5-ethynyl-2′-deoxyuridine; *ERK1/2:* extracellular signal-regulated kinases 1 and 2; *FAK*: focal adhesion kinase; FC: flow cytometry; FTL: ferritin light chain; FLIM: fluorescence lifetime imaging microscopy; GDNF: glial cell line-derived neurotrophic factor; GFAP: glial fibrillary acidic protein; GFRA1: GDNF family receptor alpha 1; GFP: green fluorescent protein; GFP-expressing GSCs: GFP-expressing glioblastoma stem cells; GPCs: glioma-propagating cells; *GPX8*: glutathione peroxidase 8; GR50: growth rate inhibition 50%; HCMV-BL: human betaherpesvirus 5 strain BL; HCMV-DB: human betaherpesvirus 5 strain DB; Hoechst: Hoechst dye; HOPX: HOP homeobox; IC50: half maximal inhibitory concentration; iPSC: induced pluripotent stem cell (from patient bone marrow-derived mononuclear cells); iPSCs: induced pluripotent stem cells; ISGs: interferon-stimulated genes; ITGA5: integrin subunit alpha 5; KO: knockout; LC3-I: microtubule-associated protein 1A/1B-light chain 3 (form I); LC3-II: microtubule-associated protein 1A/1B-light chain 3 (form II); LC3β: microtubule-associated protein 1A/1B-light chain 3 beta; LDH: lactate dehydrogenase; MAP3K1: mitogen-activated protein kinase kinase kinase 1; MDM2: MDM2 proto-oncogene; MES: mesenchymal; MES-like: mesenchymal-like; *MGMT:* O6-methylguanine-DNA methyltransferase; MMP: mitochondrial membrane potential; MSC: mesenchymal stem/stromal cell; *MSH6*: MutS homolog 6; MS-PCR: methylation-specific PCR; µM: micromolar; NANOG: Nanog homeobox; *NF-κB:* nuclear factor kappa B; NO: nitric oxide; NPC-like: neural progenitor cell-like; *OCT4*: octamer-binding transcription factor 4; *PARP:* poly(ADP-ribose) polymerase 1; PDN: neurospheres; PDO: patient-derived organoids; PDPN: podoplanin; PEG-4MAL: four-arm polyethylene glycol maleimide; PEG-Ac: polyethylene glycol acrylate; PEGDA: polyethylene glycol diacrylate; PI: propidium iodide; Polκ KO: DNA polymerase kappa knockout; *PTEN*: phosphatase and tensin homolog; ROS: reactive oxygen species; RT-qPCR: quantitative reverse transcription PCR; SATB2: special AT-rich sequence-binding protein 2; SFE: sphere-forming efficiency; shMEOX2 GLICO: MEOX2 knockdown in glioma context; *SNAIL2*: snail family transcriptional repressor 2; *SPP1:* secreted phosphoprotein 1; *SQSTM1/p62*: sequestosome 1; *STAT1:* signal transducer and activator of transcription 1; TLS: translesion synthesis; TMZ: temozolomide; TTPCCs: tumor-tolerant persister cancer cells; *TRPM7:* transient receptor potential melastatin 7; TS: thymidylate synthase; ULA: ultra-low adhesion; WT: wild type; 3D-OOAC: three-dimensional osteogenesis-angiogenesis coupling model; 3D-SUSP: three-dimensional suspension model. cell lines: A-172: brain glioblastoma cell line; BT169: brain glioblastoma cell line; BT379: patient-derived glioblastoma stem-like cells; BTSC233: primary glioblastoma stem-like cell line; CEGBCs-BL: CMV-elicited glioblastoma cells infected with HCMV-BL; CEGBCs-DB: CMV-elicited glioblastoma cells infected with HCMV-DB; DK-MG: glioblastoma multiforme cell line; DYR0100: foreskin-derived human induced pluripotent stem cells (iPSCs); G55T2: anaplastic astrocytoma cell line; GBM: glioblastoma; GBM1: glioblastoma cell line; GDNF KO: glial cell line-derived neurotrophic factor knockout (GFRA1-related); H4: neuroglioma cell line; HEK-293T: human embryonic kidney 293T cells; HK301: patient-derived glioblastoma cells; HMC3: human microglial cell line; HUVEC: human umbilical vein endothelial cells; JHH520: patient-derived glioblastoma cells; LN-18: glioblastoma cell line (right temporal lobe); LN-229: glioblastoma cell line (right frontal parieto-occipital cortex); LN-229R: glioblastoma cell line (TMZ-resistant, HSD52 knockout); NCH644: glioblastoma stem-like cell line; PDM-140: primary glioblastoma cells; PDOs: patient-derived glioblastoma cells; SF188: patient-derived temporal lobe tumor cell line; T98G: glioblastoma multiforme cell line; TDS: tumor-derived supernatants; U-118MG: glioblastoma grade IV cell line; U-138MG: glioblastoma grade IV cell line; U-251MG: glioblastoma multiforme grade IV cell line; U-251MG TLS Polκ KO: glioblastoma cell line with DNA polymerase kappa knockout (translesion synthesis); U-251MG WT: glioblastoma multiforme grade IV wild-type cell line; U-373MG: glioblastoma astrocytoma cell line; U-87MG: glioblastoma cell line; U-87MG-RFP-Luc: U-87MG cells transduced with lentiviral bicistronic vector expressing RFP and luciferase; U87-ZsGreen: U-87MG cells expressing ZsGreen fluorescent protein; U3054: patient-derived glioblastoma cells.

Abbas, S., Singh, S. K., Saxena, A. K., Tiwari, S., Sharma, L. K., and Tiwari, M. (2020). Role of autophagy in regulation of glioma stem cells population during therapeutic stress. *J Stem Cells Regen Med* 16, 80–89. doi: 10.46582/jsrm.1602012

Avci, N. G., Ebrahimzadeh-Pustchi, S., Akay, Y. M., Esquenazi, Y., Tandon, N., Zhu, J.-J., et al. (2020). NF-κB inhibitor with Temozolomide results in significant apoptosis in glioblastoma via the NF-κB(p65) and actin cytoskeleton regulatory pathways. *Sci Rep* 10, 13352. doi: 10.1038/s41598-020-70392-5

Avenel, I. C. N., Ewald, J. D., Ariey-Bonnet, J., Kristensen, I. H., Petterson, S. A., Thesbjerg, M. N., et al. (2024). GDNF/GFRA1 signaling contributes to chemo- and radioresistance in glioblastoma. *Sci Rep* 14, 17639. doi: 10.1038/s41598-024-68626-x

Bach, C., Glasow, A., Baran-Schmidt, R., Oppermann, H., Bach, C., Meixensberger, J., et al. (2024). Rapid and reproducible generation of glioblastoma spheroids for high-throughput drug screening. *Front Bioeng Biotechnol* 12, 1471012. doi: 10.3389/fbioe.2024.1471012

Basso, J., Matos, A. M., Ghavami, S., Fortuna, A., Vitorino, R., and Vitorino, C. (2024). Are we better together? Addressing a combined treatment of pitavastatin and temozolomide for brain cancer. *Eur J Pharmacol* 985, 177087. doi: 10.1016/j.ejphar.2024.177087

Bellavita, R., Barra, T., Braccia, S., Prisco, M., Valiante, S., Lombardi, A., et al. (2025). Engineering Multifunctional Peptide-Decorated Nanofibers for Targeted Delivery of Temozolomide across the Blood-Brain Barrier. *Mol Pharm* 22, 1920–1938. doi: 10.1021/acs.molpharmaceut.4c01125

Brown, J. M. C., Zaben, M., Ormonde, C., Sharouf, F., Spencer, R., Bhatt, H., et al. (2023). A high-density 3-dimensional culture model of human glioblastoma for rapid screening of therapeutic resistance. *Biochem Pharmacol* 208, 115410. doi: 10.1016/j.bcp.2023.115410

Bruns, J., Egan, T., Mercier, P., and Zustiak, S. P. (2023). Glioblastoma spheroid growth and chemotherapeutic responses in single and dual-stiffness hydrogels. *Acta Biomater* 163, 400–414. doi: 10.1016/j.actbio.2022.05.048

Cai, X., Refaat, A., Gan, P.-Y., Fan, B., Yu, H., Thang, S. H., et al. (2024). Angiopep-2-Functionalized Lipid Cubosomes for Blood-Brain Barrier Crossing and Glioblastoma Treatment. *ACS Appl Mater Interfaces* 16, 12161–12174. doi: 10.1021/acsami.3c14709

Chantaravisoot, N., Sanookpan, K., Wattanathamsan, O., Bootsri, R., Banlue, T., Chuenjit, C., et al. (2025). Targeting glioblastoma multiforme cells with pharmacological ascorbate: Disrupting DNA damage response and mTOR cascades via extracellular H2O2. *Free Radic Biol Med* 237, 326–343. doi: 10.1016/j.freeradbiomed.2025.06.008

Che, J., DePalma, T. J., Sivakumar, H., Mezache, L. S., Tallman, M. M., Venere, M., et al. (2023). αCT1 peptide sensitizes glioma cells to temozolomide in a glioblastoma organoid platform. *Biotechnol Bioeng* 120, 1108–1119. doi: 10.1002/bit.28313

Chuang, H.-Y., Cheng, C.-H., Guo, F., Yadav, V. K., Fong, I.-H., Lin, J.-W., et al. (2025). Super-enhancer PPP1R15B/EIF2A axis characterizes aggressive drug-tolerant persister cells in Glioblastoma. *Biomed Pharmacother* 189, 118309. doi: 10.1016/j.biopha.2025.118309

Ciechomska, I. A., Gielniewski, B., Wojtas, B., Kaminska, B., and Mieczkowski, J. (2020). EGFR/FOXO3a/BIM signaling pathway determines chemosensitivity of BMP4-differentiated glioma stem cells to temozolomide. *Exp Mol Med* 52, 1326–1340. doi: 10.1038/s12276-020-0479-9

Clancy, A., Chen, D., Bruns, J., Nadella, J., Stealey, S., Zhang, Y., et al. (2022). Hydrogel-based microfluidic device with multiplexed 3D in vitro cell culture. *Sci Rep* 12, 17781. doi: 10.1038/s41598-022-22439-y

Colpan, R. D., Ward, E. B., Zhang, D., Ling, B., Iqbal, U., Moreno, M., et al. (2025). Co-encapsulation of temozolomide and PbS quantum dots in apoferritin for transferrin receptor 1 targeting, imaging and treatment of glioblastoma. *Nanoscale Adv* 7, 6808–6818. doi: 10.1039/d5na00557d

Cui, L., Perini, G., Minopoli, A., Palmieri, V., De Spirito, M., and Papi, M. (2025). Plant-derived extracellular vesicles as a natural drug delivery platform for glioblastoma therapy: A dual role in preserving endothelial integrity while modulating the tumor microenvironment. *Int J Pharm X* 10, 100349. doi: 10.1016/j.ijpx.2025.100349

Datta, P., Lee, N. S., Moolayadukkam, S., Sahu, R. P., Yu, X., Guo, T., et al. (2024). In Vitro Sonodynamic Therapy Using a High Throughput 3D Glioblastoma Spheroid Model with 5-ALA and TMZ Sonosensitizers. *Adv Healthc Mater* 13, e2402877. doi: 10.1002/adhm.202402877

Davy, M., Genest, L., Legrand, C., Pelouin, O., Froget, G., Castagné, V., et al. (2023). Evaluation of Temozolomide and Fingolimod Treatments in Glioblastoma Preclinical Models. *Cancers (Basel)* 15, 4478. doi: 10.3390/cancers15184478

Dey, S., Mathur, P., Mukherjee, S., Chowdhury, R., Majumder, S., Roy, A., et al. (2025). Repurposing of CNS accumulating drugs Gemfibrozil and Doxylamine for enhanced sensitization of glioblastoma cells through modulation of autophagy. *Sci Rep* 15, 20560. doi: 10.1038/s41598-025-05054-5

Díaz Méndez, A. B., Di Giuliani, M., Sacconi, A., Tremante, E., Lulli, V., Di Martile, M., et al. (2025). Androgen receptor inhibition sensitizes glioblastoma stem cells to temozolomide by the miR-1/miR-26a-1/miR-487b signature mediated WT1 and FOXA1 silencing. *Cell Death Discov* 11, 248. doi: 10.1038/s41420-025-02517-6

Dragoj, M., Stojkovska, J., Stanković, T., Dinić, J., Podolski-Renić, A., Obradović, B., et al. (2021). Development and Validation of a Long-Term 3D Glioblastoma Cell Culture in Alginate Microfibers as a Novel Bio-Mimicking Model System for Preclinical Drug Testing. *Brain Sci* 11, 1025. doi: 10.3390/brainsci11081025

El Baba, R., Pasquereau, S., Haidar Ahmad, S., Monnien, F., Abad, M., Bibeau, F., et al. (2023). EZH2-Myc driven glioblastoma elicited by cytomegalovirus infection of human astrocytes. *Oncogene* 42, 2031–2045. doi: 10.1038/s41388-023-02709-3

Erthal, L. C. S., Shi, Y., Sweeney, K. J., Gobbo, O. L., and Ruiz-Hernandez, E. (2023). Nanocomposite formulation for a sustained release of free drug and drug-loaded responsive nanoparticles: an approach for a local therapy of glioblastoma multiforme. *Sci Rep* 13, 5094. doi: 10.1038/s41598-023-32257-5

Fabro, F., Kannegieter, N. M., de Graaf, E. L., Queiroz, K., Lamfers, M. L. M., Ressa, A., et al. (2022). Novel kinome profiling technology reveals drug treatment is patient and 2D/3D model dependent in glioblastoma. *Front Oncol* 12, 1012236. doi: 10.3389/fonc.2022.1012236

Gao, W., Huang, J., Deng, K., Lin, X., Long, X., Li, X., et al. (2026). Endothelial cells sense temozolomide resistance to facilitate monocyte-derived macrophage infiltration in glioblastoma. *Drug Resist Updat* 84, 101329. doi: 10.1016/j.drup.2025.101329

García-Sáez, C., Alonso-Marañón, J., García-Puga, M., Rubio-Zulaika, A., de Goñi-Garcia, I., Blázquez, L., et al. (2026). 3D heterotypic models of glioblastoma reveal the impact of microglia on cellular organization and the production of a distinct secretome. *Sci Rep* 16, 7246. doi: 10.1038/s41598-026-37395-0

Glia, A., Deliorman, M., Sukumar, P., and Qasaimeh, M. A. (2025). Spheromatrix: a paper-based platform for scalable 3D tumor model generation, cryopreservation, and high-throughput drug assessment. *Microsyst Nanoeng* 11, 219. doi: 10.1038/s41378-025-01077-0

Gozdz, A., Wojtaś, B., Szpak, P., Szadkowska, P., Czernicki, T., Marchel, A., et al. (2022). Preservation of the Hypoxic Transcriptome in Glioblastoma Patient-Derived Cell Lines Maintained at Lowered Oxygen Tension. *Cancers (Basel)* 14, 4852. doi: 10.3390/cancers14194852

Han, S., Kim, S., Chen, Z., Shin, H. K., Lee, S.-Y., Moon, H. E., et al. (2020). 3D Bioprinted Vascularized Tumour for Drug Testing. *Int J Mol Sci* 21, 2993. doi: 10.3390/ijms21082993

Hill, L., Bruns, J., and Zustiak, S. P. (2021). Hydrogel matrix presence and composition influence drug responses of encapsulated glioblastoma spheroids. *Acta Biomater* 132, 437–447. doi: 10.1016/j.actbio.2021.05.005

Hincapié-Arias, E., Moughty-Cueto, C., Zaloff-Dakoff, J. M., Mazzon, A. J., Gómez-Escalante, J. I., Merenzon, M. A., et al. (2025). NO/iNOS axis impact on glioma stem cells and temozolomide resistance. *J Mol Med (Berl)* 104, 16. doi: 10.1007/s00109-025-02609-x

Hwang, J. W., Loisel-Duwattez, J., Desterke, C., Latsis, T., Pagliaro, S., Griscelli, F., et al. (2020). A novel neuronal organoid model mimicking glioblastoma (GBM) features from induced pluripotent stem cells (iPSC). *Biochim Biophys Acta Gen Subj* 1864, 129540. doi: 10.1016/j.bbagen.2020.129540

Khotchawan, W., Lorthongpanich, C., Kheolamai, P., Sathornsumetee, S., and Issaragrisil, S. (2025). Recovery of glioblastoma cells derived from patients after a decade of cryopreservation for drug response studies. *Sci Rep* 15, 39424. doi: 10.1038/s41598-025-22908-0

Lam, M. S., Aw, J. J., Tan, D., Vijayakumar, R., Lim, H. Y. G., Yada, S., et al. (2023). Unveiling the Influence of Tumor Microenvironment and Spatial Heterogeneity on Temozolomide Resistance in Glioblastoma Using an Advanced Human In Vitro Model of the Blood-Brain Barrier and Glioblastoma. *Small* 19, e2302280. doi: 10.1002/smll.202302280

Lee, G., Kim, S. J., Choi, Y., Park, J., and Park, J.-K. (2024). Bioprinting of a multi-composition array to mimic intra-tumor heterogeneity of glioblastoma for drug evaluation. *Microsyst Nanoeng* 10, 186. doi: 10.1038/s41378-024-00843-w

Maity, S., Jewell, C., Yilgor, C., Kawakita, S., Sharma, S., Gomez, A., et al. (2025). Deciphering pericyte-induced temozolomide resistance in glioblastoma with a 3D microphysiological system mimicking the biomechanical properties of brain tissue. *Acta Biomater* 200, 202–217. doi: 10.1016/j.actbio.2025.05.038

Majc, B., Habič, A., Malavolta, M., Vittori, M., Porčnik, A., Bošnjak, R., et al. (2024). Patient-derived tumor organoids mimic treatment-induced DNA damage response in glioblastoma. *iScience* 27, 110604. doi: 10.1016/j.isci.2024.110604

Mannino, D., Calcaterra, E., Scuderi, S. A., Caffo, M., De Luca, F., Catalfamo, A., et al. (2025). Troxerutin Potentiated Temozolomide Induced Antitumor Effect in 2D and 3D Glioblastoma Models. *J Cell Mol Med* 29, e70862. doi: 10.1111/jcmm.70862

Mendieta, M., Avci, N. G., Pandurangi, R., Akay, Y. M., and Akay, M. (2023). Targeted Sensitization of Glioblastoma Multiforme Using AAAPT Technology. *IEEE Open J Eng Med Biol* 4, 251–258. doi: 10.1109/OJEMB.2023.3336181

Mendieta, M., Bandegi, M., Biltekin, E., Akay, Y. M., Ozpolat, B., and Akay, M. (2025a). MiR 329/449 Suppresses Cell Proliferation, Migration and Synergistically Sensitizes GBM to TMZ by Inhibiting Src/FAK, NF-kB, and Cyclin D1 Activity. *Int J Mol Sci* 26, 5533. doi: 10.3390/ijms26125533

Mendieta, M., Hatami, M., Singh, M., Saeidi Fard, S., Dehshiri, M., Schill, A., et al. (2025b). Non-Invasive Measurement of Elasticity in Glioblastoma Multiforme Validates Decreased TMZ Sensitivity in Astrocyte Co-Culture. *IEEE Open J Eng Med Biol* 6, 287–295. doi: 10.1109/OJEMB.2025.3528194

Moore, Z., Storey, C., Brown, D. V., Valkovic, A., Spiteri, M., Pignatelli, J. F., et al. (2026). Patient-derived organoids and neurospheres recapitulate salient features of primary tumor heterogeneity. *Neurooncol Adv* 8, vdaf247. doi: 10.1093/noajnl/vdaf247

Morelli, M., Lessi, F., Franceschi, S., Ferri, G., Giacomarra, M., Menicagli, M., et al. (2024). Exploring Regorafenib Responsiveness and Uncovering Molecular Mechanisms in Recurrent Glioblastoma Tumors through Longitudinal In Vitro Sampling. *Cells* 13, 487. doi: 10.3390/cells13060487

Na, M. K., Oh, Y., Lee, D., Park, J., Yoon, S.-J., Yoo, J., et al. (2025). Comparison of the biological characteristics of glioblastoma tumorspheres obtained from fresh and cryopreserved glioblastoma tissues. *J Neurooncol* 174, 191–206. doi: 10.1007/s11060-025-05052-7

Nakod, P. S., Kondapaneni, R. V., Edney, B., Kim, Y., and Rao, S. S. (2022). The impact of temozolomide and lonafarnib on the stemness marker expression of glioblastoma cells in multicellular spheroids. *Biotechnol Prog* 38, e3284. doi: 10.1002/btpr.3284

Oliveira, L. S., Oliveira-Silva, J. M., Almeida-Souza, H. O., Martins, M. M., Chiminazo, C. B., Fonseca, R., et al. (2025). HDAC6 inhibition through WT161 synergizes with temozolomide, induces apoptosis, reduces cell motility, and decreases β-catenin levels in glioblastoma cells. *Invest New Drugs* 43, 223–242. doi: 10.1007/s10637-025-01508-9

Oliver, L., Álvarez-Arenas, A., Salaud, C., Jiménez-Sanchez, J., Calvo, G. F., Belmonte-Beitia, J., et al. (2023). A Simple 3D Cell Culture Method for Studying the Interactions between Human Mesenchymal Stromal/Stem Cells and Patients Derived Glioblastoma. *Cancers (Basel)* 15, 1304. doi: 10.3390/cancers15041304

Oliver, L., Landais, Y., Gratas, C., Cartron, P.-F., Paris, F., Heymann, D., et al. (2024). Transcriptional landscape of the interaction of human Mesenchymal Stem Cells with Glioblastoma in bioprinted co-cultures. *Stem Cell Res Ther* 15, 424. doi: 10.1186/s13287-024-04022-6

Oraiopoulou, M.-E., Tzamali, E., Psycharakis, S. E., Tzedakis, G., Makatounakis, T., Manolitsi, K., et al. (2024). The Temozolomide–Doxorubicin paradox in Glioblastoma in vitro–in silico preclinical drug-screening. *Scientific Reports* 14, 3759. doi: 10.1038/s41598-024-53684-y

Park, H. H., Park, J., Cho, H. J., Shim, J.-K., Moon, J. H., Kim, E. H., et al. (2021). Combinatorial Therapeutic Effect of Inhibitors of Aldehyde Dehydrogenase and Mitochondrial Complex I, and the Chemotherapeutic Drug, Temozolomide against Glioblastoma Tumorspheres. *Molecules* 26, 282. doi: 10.3390/molecules26020282

Payan, B. A., De Leon, A. C. D., Anand, T., Thompson, G. B., Krishnamurthy, V. V., Mora-Boza, A., et al. (2026). Hydrogel Microdroplet Based Glioblastoma Drug Screening Platform. *J Biomed Mater Res A* 114, e70043. doi: 10.1002/jbma.70043

Peng, T., Ma, X., Hua, W., Wang, C., Chu, Y., Sun, M., et al. (2025). Individualized patient tumor organoids faithfully preserve human brain tumor ecosystems and predict patient response to therapy. *Cell Stem Cell* 32, 652-669.e11. doi: 10.1016/j.stem.2025.01.002

Pérez-Aliacar, M., Ayensa-Jiménez, J., Ranđelović, T., Ochoa, I., and Doblaré, M. (2024). Modelling glioblastoma resistance to temozolomide. A mathematical model to simulate cellular adaptation in vitro. *Comput Biol Med* 180, 108866. doi: 10.1016/j.compbiomed.2024.108866

Phour, J., and Vassella, E. (2024). Methods in cancer research: Assessing therapy response of spheroid cultures by life cell imaging using a cost-effective live-dead staining protocol. *Biol Methods Protoc* 9, bpae060. doi: 10.1093/biomethods/bpae060

Potthoff, A.-L., Hsieh, M.-C., Melhem, A., Ng, S. S., Pregler, B. E. F., Vieregge, A., et al. (2025). Flow cytometry protocol for cell death analysis in glioblastoma organoids: A technical note. *PLoS One* 20, e0327660. doi: 10.1371/journal.pone.0327660

Pun, S., Prakash, A., Demaree, D., Krummel, D. P., Sciumè, G., Sengupta, S., et al. (2024). Rapid Biofabrication of an Advanced Microphysiological System Mimicking Phenotypical Heterogeneity and Drug Resistance in Glioblastoma. *Adv Healthc Mater* 13, e2401876. doi: 10.1002/adhm.202401876

Pustchi, S. E., Avci, N. G., Akay, Y. M., and Akay, M. (2020). Astrocytes Decreased the Sensitivity of Glioblastoma Cells to Temozolomide and Bay 11-7082. *Int J Mol Sci* 21, 7154. doi: 10.3390/ijms21197154

Qu, S., Qi, S., Zhang, H., Li, Z., Wang, K., Zhu, T., et al. (2023). Albumin-bound paclitaxel augment temozolomide treatment sensitivity of glioblastoma cells by disrupting DNA damage repair and promoting ferroptosis. *J Exp Clin Cancer Res* 42, 285. doi: 10.1186/s13046-023-02843-6

Rampazzo, E., Persano, L., Karim, N., Hodgking, G., Pinto, R., Casciati, A., et al. (2024). On the effects of 30.5 GHz sinusoidal wave exposure on glioblastoma organoids. *Front Oncol* 14, 1307516. doi: 10.3389/fonc.2024.1307516

Ribeiro, D. L., Latancia, M. T., de Souza, I., Ariwoola, A.-B. A., Mendes, D., Rocha, C. R. R., et al. (2024). Temozolomide resistance mechanisms: unveiling the role of translesion DNA polymerase kappa in glioblastoma spheroids in vitro. *Biosci Rep* 44, BSR20230667. doi: 10.1042/BSR20230667

Riess, C., Koczan, D., Schneider, B., Linke, C., Del Moral, K., Classen, C. F., et al. (2021). Cyclin-dependent kinase inhibitors exert distinct effects on patient-derived 2D and 3D glioblastoma cell culture models. *Cell Death Discov* 7, 54. doi: 10.1038/s41420-021-00423-1

Rodrigues-Junior, D. M., Raminelli, C., Hassanie, H., Trossini, G. H. G., Perecim, G. P., Caja, L., et al. (2022). Aporphine and isoquinoline derivatives block glioblastoma cell stemness and enhance temozolomide cytotoxicity. *Sci Rep* 12, 21113. doi: 10.1038/s41598-022-25534-2

Russo, M., Valentini, E., Aliperti, V., Copparoni, F., Linkous, A., Montaldo, C., et al. (2026). MEOX2 enhances DNA repair and therapy resistance in Glioblastoma stem-like cells via PARP1 interaction. *Cancer Letters* 642, 218284. doi: 10.1016/j.canlet.2026.218284

Sambamoorthy, I. A., Arumugam, B., Manikandan, C., and Jaiswal, A. K. (2026). 3D-Printed Scaffold-Based Glioblastoma Spheroid In Vitro Model for Drug Screening Application. *Ann Biomed Eng* 54, 117–133. doi: 10.1007/s10439-025-03892-y

Samiei, E., Seyfoori, A., Toyota, B., Ghavami, S., and Akbari, M. (2020). Investigating Programmed Cell Death and Tumor Invasion in a Three-Dimensional (3D) Microfluidic Model of Glioblastoma. *Int J Mol Sci* 21, 3162. doi: 10.3390/ijms21093162

Scheffel, T. B., Rockenbach, L., Cruz, F. F., Kist, L. W., Bogo, M. R., Scholl, J. N., et al. (2022). Inhibition of ATP hydrolysis as a key regulator of temozolomide resistance and migratory phenotype of glioblastoma cells. *Biochemical and Biophysical Research Communications* 601, 24–30. doi: 10.1016/j.bbrc.2022.02.062

Scuderi, S. A., Ardizzone, A., Mannino, D., Palermo, N., De Luca, F., Catalfamo, A., et al. (2026). Modulation of Wnt/β-Catenin Pathway by Aesculus hippocastanum Extract Enhances Temozolomide Sensitivity in Glioblastoma Cells. *J Cell Mol Med* 30, e70979. doi: 10.1111/jcmm.70979

Seyfoori, A., Liu, K., Caruncho, H. J., Walter, P. B., and Akbari, M. (2025). Tumoroid-On-a-Plate (ToP): Physiologically Relevant Cancer Model Generation and Therapeutic Screening. *Adv Healthc Mater* 14, e2402060. doi: 10.1002/adhm.202402060

Sha, X., Li, J., Fang, Y., Feng, J., Feng, Y., Wang, J., et al. (2025). Enhanced tumor suppression in patient-derived temozolomide-resistant glioblastoma cells using a combination treatment of Olaparib and FK866. *BMC Cancer* 25, 1174. doi: 10.1186/s12885-025-14568-0

Shaw, P., Kumar, N., Privat-Maldonado, A., Smits, E., and Bogaerts, A. (2021). Cold Atmospheric Plasma Increases Temozolomide Sensitivity of Three-Dimensional Glioblastoma Spheroids via Oxidative Stress-Mediated DNA Damage. *Cancers (Basel)* 13, 1780. doi: 10.3390/cancers13081780

Shojaei, S., Koleini, N., Samiei, E., Aghaei, M., Cole, L. K., Alizadeh, J., et al. (2020). Simvastatin increases temozolomide-induced cell death by targeting the fusion of autophagosomes and lysosomes. *FEBS J* 287, 1005–1034. doi: 10.1111/febs.15069

Singh, S. K., Abbas, S., Saxena, A. K., Tiwari, S., Sharma, L. K., and Tiwari, M. (2020). Critical role of three-dimensional tumorsphere size on experimental outcome. *Biotechniques* 69, 333–338. doi: 10.2144/btn-2020-0081

Sørensen, M. D., Olsen, R. F. S., Burton, M., Kavan, S., Petterson, S. A., Thomassen, M., et al. (2024). Microglia induce an interferon-stimulated gene expression profile in glioblastoma and increase glioblastoma resistance to temozolomide. *Neuropathol Appl Neurobiol* 50, e13016. doi: 10.1111/nan.13016

Sousa, N., Geiß, C., Bindila, L., Lieberwirth, I., Kim, E., and Régnier-Vigouroux, A. (2023). Targeting sphingolipid metabolism with the sphingosine kinase inhibitor SKI-II overcomes hypoxia-induced chemotherapy resistance in glioblastoma cells: effects on cell death, self-renewal, and invasion. *BMC Cancer* 23, 762. doi: 10.1186/s12885-023-11271-w

Sun, N., Chen, Q., Chen, H., Sun, P., Liu, Y., Song, D., et al. (2025). A novel nuclear RNA HSD52 scaffolding NONO/SFPQ complex modulates DNA damage repair to facilitate temozolomide resistance. *Neuro Oncol* 27, 963–978. doi: 10.1093/neuonc/noae272

Sundar, S. J., Shakya, S., Barnett, A., Wallace, L. C., Jeon, H., Sloan, A., et al. (2022). Three-dimensional organoid culture unveils resistance to clinical therapies in adult and pediatric glioblastoma. *Transl Oncol* 15, 101251. doi: 10.1016/j.tranon.2021.101251

Tafech, A., Jacquet, P., Beaujean, C., Fertin, A., Usson, Y., and Stéphanou, A. (2023). Characterization of the Intracellular Acidity Regulation of Brain Tumor Cells and Consequences for Therapeutic Optimization of Temozolomide. *Biology (Basel)* 12, 1221. doi: 10.3390/biology12091221

Tehrani, G. A., Kubick, R. N., Zarodniuk, M., and Datta, M. (2025). Histone deacetylase inhibitors sensitize glioblastoma models to temozolomide and reprogram immunosuppressive myeloid cells. *Sci Rep* 15, 36804. doi: 10.1038/s41598-025-20749-5

Vargas-Toscano, A., Nickel, A.-C., Li, G., Kamp, M. A., Muhammad, S., Leprivier, G., et al. (2020). Rapalink-1 Targets Glioblastoma Stem Cells and Acts Synergistically with Tumor Treating Fields to Reduce Resistance against Temozolomide. *Cancers (Basel)* 12, 3859. doi: 10.3390/cancers12123859

Verduin, M., Hoosemans, L., Vanmechelen, M., van Heumen, M., Piepers, J. A. F., Astuti, G., et al. (2023). Patient-derived glioblastoma organoids reflect tumor heterogeneity and treatment sensitivity. *Neurooncol Adv* 5, vdad152. doi: 10.1093/noajnl/vdad152

Wagner, K. K., Heinze, A., Zenz, T., Yool, A. J., Franke, H., and Aigner, A. (2026). Xenograft tissue slice tandem co-cultures are a highly specific model to selectively analyze drug inhibitory effects on glioblastoma invasion. *J Biol Chem* 302, 110986. doi: 10.1016/j.jbc.2025.110986

Wanigasekara, J., Mondala, J. R. M., Cullen, P. J., Tiwari, B. K., Kinsella, G. K., and Curtin, J. F. (2025). Ultrasound-Mediated Drug Diffusion, Uptake, and Cytotoxicity in a Glioblastoma 3D Tumour Sphere Model. *Cells* 14, 886. doi: 10.3390/cells14120886

Watanabe, F., Hollingsworth, E. W., Bartley, J. M., Wisehart, L., Desai, R., Hartlaub, A. M., et al. (2024). Patient-derived organoids recapitulate glioma-intrinsic immune program and progenitor populations of glioblastoma. *PNAS Nexus* 3, pgae051. doi: 10.1093/pnasnexus/pgae051

Wu, S., Xue, S., Tang, Y., Zhao, W., Zheng, M., Cheng, Z., et al. (2024). Mitogen-activated protein kinase kinase kinase 1 facilitates the temozolomide resistance and migration of GBM via the MEK/ERK signalling. *J Cell Mol Med* 28, e70173. doi: 10.1111/jcmm.70173

Xiao, W., Wang, S., Zhang, R., Sohrabi, A., Yu, Q., Liu, S., et al. (2020). Bioengineered scaffolds for 3D culture demonstrate extracellular matrix-mediated mechanisms of chemotherapy resistance in glioblastoma. *Matrix Biol* 85–86, 128–146. doi: 10.1016/j.matbio.2019.04.003

Yadav, A., Alnakhli, A., Vemana, H. P., Bhutkar, S., Muth, A., and Dukhande, V. V. (2022). Repurposing an Antiepileptic Drug for the Treatment of Glioblastoma. *Pharm Res* 39, 2871–2883. doi: 10.1007/s11095-022-03399-4

Ye, R., Zhang, W., Zhang, H., Qu, S., Xu, J., Xu, R., et al. (2025). The HBP Pathway Inhibitor FR054 Enhances Temozolomide Sensitivity in Glioblastoma Cells by Promoting Ferroptosis and Inhibiting O-GlcNAcylation. *CNS Neurosci Ther* 31, e70546. doi: 10.1111/cns.70546

Yu, X., Wang, M., Chen, W., Jing, J., and Yu, H. (2026). TEAD4-driven GPX8 promotes temozolomide resistance in glioma by facilitating CTHRC1 expression to suppress mitochondrial oxidative stress. *Naunyn Schmiedebergs Arch Pharmacol* 399, 9101–9121. doi: 10.1007/s00210-026-04992-9

Yue, W., Wang, T., Xie, L., and Shen, J. (2024). An injectable in situ hydrogel platform for sustained drug release against Glioblastoma. *J Drug Deliv Sci Technol* 95, 105527. doi: 10.1016/j.jddst.2024.105527

Zirjacks, L., Stransky, N., Klumpp, L., Prause, L., Eckert, F., Zips, D., et al. (2021). Repurposing Disulfiram for Targeting of Glioblastoma Stem Cells: An In Vitro Study. *Biomolecules* 11, 1561. doi: 10.3390/biom11111561

Zuo, M., Zhang, S., Chen, S., He, Y., Li, J., Xiang, Y., et al. (2025). Cytokine CCL2 secreted by cancer-associated fibroblasts augments temozolomide resistance in glioblastoma through ERK1/2 signaling. *Oncogene* 44, 4657–4670. doi: 10.1038/s41388-025-03601-y
